# Single-cell RNA-sequencing reveals distinct immune cell subsets and signaling pathways in IgA nephropathy

**DOI:** 10.1186/s13578-021-00706-1

**Published:** 2021-12-11

**Authors:** Honghui Zeng, Le Wang, Jiajia Li, Siweier Luo, Qianqian Han, Fang Su, Jing Wei, Xiaona Wei, Jianping Wu, Bin Li, Jingang Huang, Patrick Tang, Chunwei Cao, Yiming Zhou, Qiongqiong Yang

**Affiliations:** 1 Guangdong Provincial Key Laboratory of Malignant Tumor Epigenetics and Gene Regulation, Guangdong-Hong Kong Joint Laboratory for RNA Medicine, Sun Yat-Sen Memorial Hospital, Sun Yat-Sen University, Guangzhou, 510120 Guangdong China; 2 Medical Research Center, Sun Yat-Sen Memorial Hospital, Sun Yat-Sen University, Guangzhou, 510120 Guangdong China; 3 Department of Nephrology, Sun Yat-Sen Memorial Hospital, Sun Yat-Sen University, Guangzhou, 510120 Guangdong China; 4 Clinical Trials Unit, The First Affiliated Hospital of Sun Yat-Sen University, Sun Yat-Sen University, Guangzhou, 510120 Guangdong China; 5 Department of Anatomical and Cellular Pathology, The Chinese University of Hong Kong, Shatin, Hong Kong, China

**Keywords:** IgA nephropathy, Single-cell RNA seq, Immune cell landscape, Natural killer cells, Monocytes, B cells

## Abstract

**Background:**

IgA nephropathy (IgAN) is the most common primary glomerulonephritis globally. Increasing evidence suggests the importance of host immunity in the development of IgAN, but its dynamics during the early stage of IgAN are still largely unclear.

**Results:**

Here we successfully resolved the early transcriptomic changes in immune cells of IgAN by conducting single-cell RNA-sequencing (scRNA-seq) with peripheral blood mononuclear cells. The differentially expressed genes (DEGs) between control and IgAN were predominantly enriched in NK cell-mediated cytotoxicity and cell killing pathways. Interestingly, we discovered that the number and cytotoxicity of NK cells are significantly reduced in IgAN patients, where both the number and marker genes of NK cells were negatively associated with the clinical parameters, including the levels of urine protein creatinine ratio (UPCR), serum galactose-deficient IgA1 and IgA. A distinctive B cell subset, which had suppressed NFκB signaling was predominantly in IgAN and positively associated with disease progression. Moreover, the DEGs of B cells were enriched in different viral infection pathways. Classical monocytes also significantly changed in IgAN and a monocyte subset expressing interferon-induced genes was positively associated with the clinical severity of IgAN. Finally, we identified vast dynamics in intercellular communications in IgAN.

**Conclusions:**

We dissected the immune landscape of IgAN at the single-cell resolution, which provides new insights in developing novel biomarkers and immunotherapy against glomerulonephritis.

**Supplementary Information:**

The online version contains supplementary material available at 10.1186/s13578-021-00706-1.

## Background

IgA nephropathy (IgAN) is the most common primary glomerulonephritis globally and is caused by the mesangial IgA immune complex deposits, leading to macroscopic hematuria with or without proteinuria [1, 2]. Approximately one-third of IgAN patients will develop the end-stage renal disease (ESRD) in two decades after the proven diagnosis by renal biopsy [3, 4]. Numerous studies indicate that IgAN is not originated from a single, but multiple hits of both genetic and environmental factors. Currently, the “four-hit” hypothesis is a well-accepted pathogenesis model that could explain the major clinical phenomena of IgAN [2, 5]. The first hit is the elevated synthesis of galactose-deficient IgA1 (gd-IgA1) by B cells. The following two hits are the production of specific IgG autoantibodies that recognize the gd-IgA1 and the formation of the immune complex containing gd-IgA1 and IgG. The last hit is the deposition of the immune complexes in glomeruli and driving mesangial hyperplasia, cytokine and chemokine overproduction, matrix expansion eventually glomerular injury. Specifically, IgA1, but not IgA2, displays a reduced content of galactose in the hinge region in the circulation as well as in the mesangial immune complex deposits. In the absence of galactose, the terminal sugar becomes N-acetylgalactosamine, where sialic acid is attached [6, 7]. The modified sugar moieties and the glycopeptides [8, 9] are recognized by anti-glycan antibodies [7, 9, 10], eventually, gd-IgA1-IgG immune complexes are formed [6, 7, 11–13]. Some of these immune complexes may escape from hepatic clearance and deposit in the renal glomeruli leading the mesangial hyperplasia and glomerular injury [7, 12–14].

The human immune system is a complex network comprised of both innate and adaptive immunity that can influence IgAN. Genetic studies show that there are a group of single nucleotide polymorphisms within loci that are related to antigen presentation (e.g., MHC), complement system (e.g. CFH, CFHR3-1, ITGAM-ITGAX), and mucosal immunity (e.g. DEFA, CARD9, VAV3) [15–18]. Various studies have indicated that B cells are crucial in IgAN pathogenesis. The activation of APRIL and BAFF pathways promotes the production of gd-IgA1 through IgA class switching recombination and contributes to IgAN development [19]. Toll-like receptor 9 (TLR9) in B cells can recognize PAMPs and/or DAMP, and induce gd-IgA1 production via APRIL and IL-6 pathway [20]. BAFF-Tg mice display mesangial immune complex deposits together with a high serum level of polymeric gd-IgA, although the structure of mouse IgA is not the same as human IgA1 [21]. Myeloid and T cells may also participate in IgAN pathogenesis. During mucosal infection, IFN-α and other cytokines secreted from myeloid cells stimulate the secretion of IFN-γ by NK cells. The increased level of IFN-γ further up-regulates BAFF production [22]. The imbalance of T cell subsets could lead to abnormal proliferation and secretion of gd-IgA1 in B cells. Complement components like C3 and C4 are also found in the gd-IgA1 immune complex and its excessive activation may be the dominant drivers of renal injury in IgAN [23, 24]. However, the origin of the gd-IgA1-secreting B and plasma cells and the expression of which transcriptomic profiles remain unelucidated. In addition, whether and how other types of immune cells participate in IgAN is also unclear. These questions immensely hinder the development of immune cell-based diagnosis and therapies for IgAN.

Single-cell RNA sequencing (scRNA-seq) is rapidly emerging as a powerful tool to explore the transcriptomic heterogeneity at the single-cell level. It has been successfully applied to establish human kidney cell atlas, examine the kidney disease pathogenesis as well as kidney development at the single-cell resolution [25–29]. Recently, a study using scRNA-seq examined kidney biopsy tissues and CD14 + monocytes from IgAN patients and revealed specific gene expression changes in kidney resident macrophages and monocytes between IgAN and control samples [30]. This study found that the activation of kidney resident macrophages in IgAN may be caused by metabolic reprograming. Moreover, the T cell cytotoxicity-related gene was markedly decreased, and CD14 + monocytes exhibited a greater interferon response in IgAN samples compared with that of control. To date, however, there is still a lack of knowledge and resources of the comprehensive single-cell transcriptomic landscape for peripheral mononuclear cells in IgAN.

Hence, we studied PBMCs from six healthy donors and ten IgAN patients using scRNA-seq technology. We have captured and analyzed a total number of 39,426 cells and annotated these cells into 15 cell clusters. Our results identified that besides CD14 + monocytes, multiple cell types, genes, and pathways were closely associated with IgAN. We also identified vast dynamics in intercellular communication networks in IgAN. By integrating our scRNA-seq results with previously reported GWAS genes and illustrating their distribution patterns in immune cells, offering directions for studying the molecular mechanism of GWAS genes in IgAN. Our results provide a comprehensive single-cell landscape and resource of peripheral immune cells in IgAN disease, which may facilitate the development of novel immune cell-based biomarkers, diagnostic techniques, and novel targeted therapies.

## Results

### scRNA-seq of PBMCs from healthy control donors and IgAN patients

**Fig. 1:**
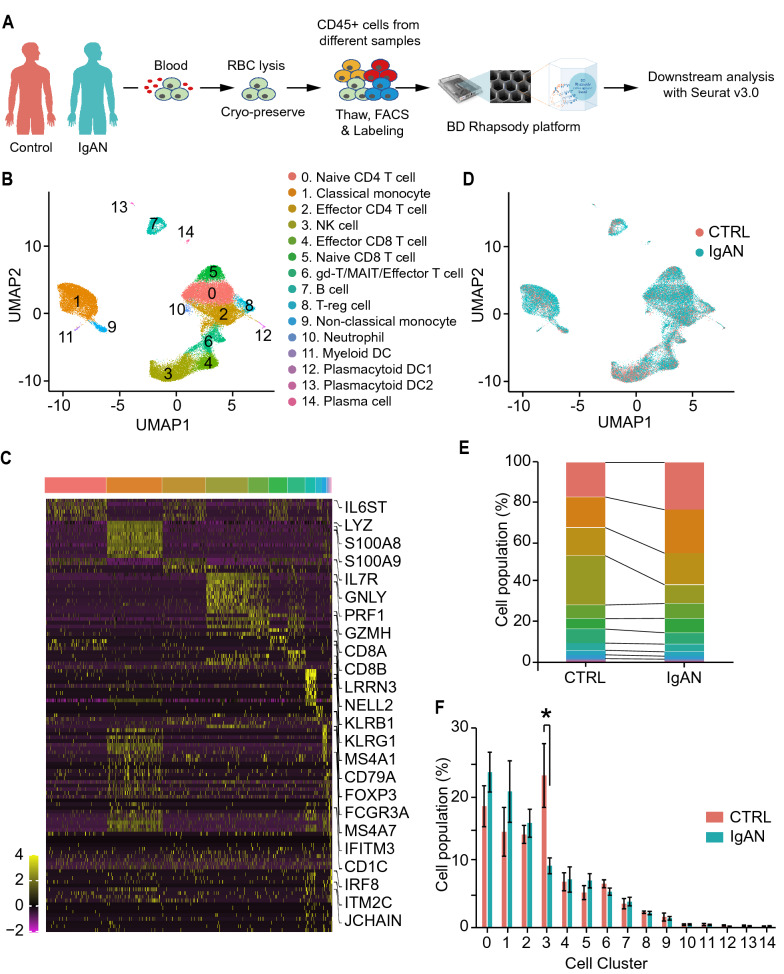
Single-cell landscape of peripheral blood mononuclear cells (PBMCs) from healthy control donors and IgAN patients. **A** Schematic flowchart of scRNA-seq experimental design of this study. Control (CTRL) n = 6; IgAN n = 10. **B**,** C** UMAP illustration of integrated scRNA-seq data from CTRL and IgAN colored by cell-type annotation (**B**) and cells of origin (**C**). **D** Heatmap of top ten marker genes of each cell cluster. **E** Stacked bar graphs of the cell-type composition in PBMCs from CTRL and IgAN. **F** Bar graphs of each cell cluster population between CTRL and IgAN. CTRL n = 6; IgAN n = 10; Mean ± SEM; *p < 0.05

To capture and understand the immune cell transcriptomic changes in the early stage of IgAN, we studied the patients with newly-diagnosed biopsy-proven IgAN. There were no significant differences among age, gender, BMI and blood pressure between healthy control donors and IgAN patients, while the patients presented higher levels of urine protein creatinine ratio (UPCR, 476.03 ± 106.18 vs. 57.39 ± 7.60 mg/g, p = 0.0011), urine red blood cell (uRBC, 53.23 ± 16.30 vs. 0.25 ± 0.11 /HPF, p = 0.0011), serum galactose-deficient IgA1 (gd-IgA1, 5876.48 ± 972.85 vs. 2372.80 ± 329.71 ng/mL, p = 0.0034), and serum IgA (3.30 ± 0.34 vs. 1.81 ± 0.19 g/L, p = 0.0020) as compared with those of healthy controls (Additional file 1: Table S1). Peripheral blood mononuclear cells (PBMCs) were collected from six control health donors (CTRL) and ten IgAN patients (IgAN), and were cryo-preserved until the days for scRNA-seq experiments. Three to five PBMC samples, at least one from healthy control donors, were labeled with different sample tags using BD Single-Cell Multiplexing kit and then pooled together for one scRNA-seq experiment (Fig. 1A and Additional file 1: Fig. S1A). Together, we have performed four scRNA-seq experiments for a total of 16 samples. The distribution plots of gene count to cell number were consistent among four scRNA-seq experiments (Additional file 1: Fig. S1B). Specifically, the numbers of RNA per cell (Additional file 1: Fig. S1C), the features of RNA per cell (Additional file 1: Fig. S1D), and the numbers of mitochondria gene per cell (Additional file 1: Fig. S1E) were consistent among all the PBMC samples regardless of their experiment batches or cells of origin. Meanwhile, we also observed a strong positive correlation (R^2^ = 0.97) between the numbers and features of RNA per cell among all the PBMC samples (Additional file 1: Fig. S1F). We then performed QC and filtered the cells of each sample with the following criteria: less than 200 or more than 2500 genes per cell, or more than 15% of reads mapping to mitochondria genes. Passed cells were used for the downstream analysis (Additional file 1: Fig. S1G). The pass rates were 62.5%, 60.2%, and 63.9% for all PBMCs, PBMCs from CTRL, and PBMCs from IgAN, respectively (Additional file 1: Fig. S1H). The final cell numbers for downstream analysis were 14,060 and 25,366 for CTRL and IgAN, respectively. To rule out the cell number-induced downstream analysis bias, we also confirmed that after QC, each sample has a fair contribution to the cells for downstream analysis (Additional file 1: Fig. S1I). The average cell percentages of CTRL and IgAN for downstream analysis were 5.8% and 6.5%, respectively.

We firstly performed dimension reduction of four scRNA-seq datasets using Uniform Manifold Approximation and Projection (UMAP) without the data integration. We found that these scRNA-seq datasets could be clustered together according to their transcriptomic profiles, although there were some degrees of variations among each scRNA-seq experiment. While after the data integration with Seurat V3.0, four scRNA-seq datasets were completely integrated and harmonized, with all 15 cell clusters presented in each scRNA-seq dataset (Additional file 1: Fig. S2A and S2B). We then performed downstream analysis after the data integration and annotated each cluster with its marker genes that were previously reported and validated (Fig. 1B and Additional file 1: Fig. S3). We have successfully captured major PBMC cell types, including naïve CD4 + T cells, naïve CD8 + T cells, effector CD4 + T cells, effector CD8 + T cells, B cells, plasma cells, NK cells, monocytes, and various DCs. We then visualized the cells in the UMAP colored by their origins: CTRL and IgAN cells were colored with red and blue, respectively (Fig. 1C). The heatmap and dot plot confirmed the distinct gene expression patterns among these 15 clusters (Fig. 1D and Additional file 1: Fig. S4). Most of the cells were evenly distributed in CTRL and IgAN, however, we observed some differences in NK, B cells, and monocytes (Fig. 1E and Additional file 1: Fig. S5A). Therefore, we compared the cell population of each cluster. Interestingly, we discovered that there was a significant decrease in the number of NK cells and a slight increase in the number of monocytes of IgAN compared with that of CTRL (Fig. 1F, and Additional file 1: Fig. S5B). Previous two studies have suggested that the decrease in number and function of NK cells were associated with the disease progression of IgAN [31, 32]. Altered monocyte gene expression patterns and numbers were found to be pathogenic factors in IgAN [33, 34]. Higher respiratory burst and metabolic reprogramming in monocytes have been linked to the pathogenesis of IgAN [30, 35]. To date, however, a comprehensive understanding of the contribution of these cells to IgAN is still lacking.

### scRNA-seq uncovered the DEGs from IgAN predominantly expressed in NK, monocytes, and B cells

**Fig. 2:**
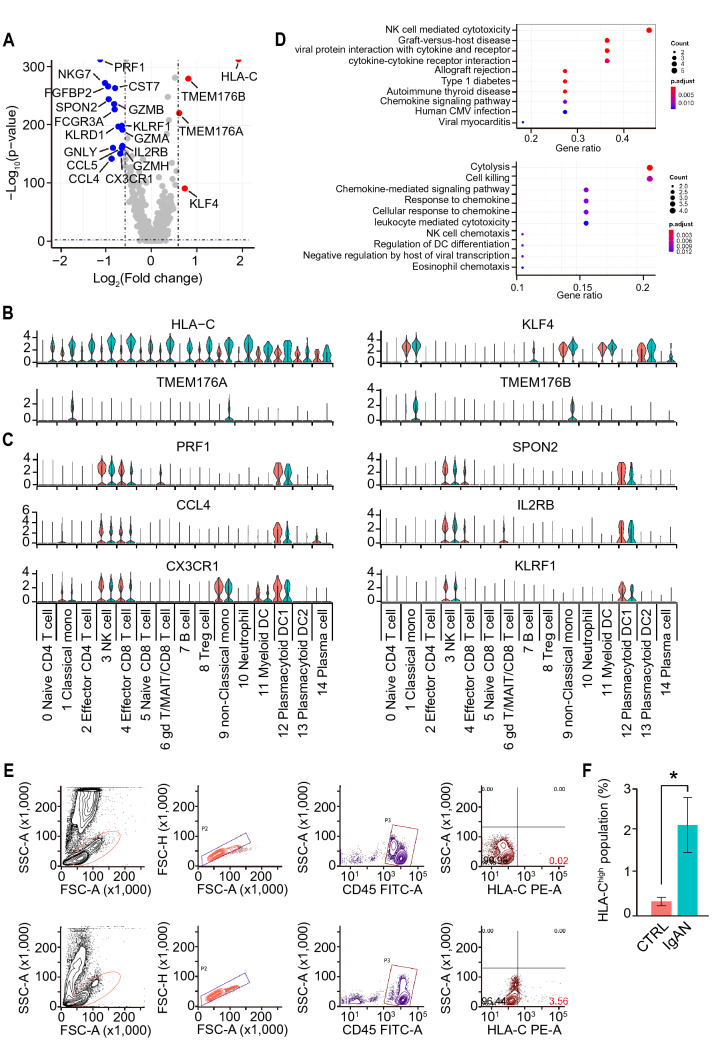
scRNA-seq discovered the differentially expressed genes (DEGs) in PBMCs from control and IgAN. **A** Volcano plot of both up- (Red circles) and down-regulated (Blue circles) DEGs in IgAN PBMCs compared with those in CTRL. **B**, **C** Violin plots of the up- (**B**) and down-regulated (**C**) DE genes in each cell cluster from CTRL (Red violin) and IgAN (Blue violin). **D** KEGG pathway (Upper) and GO enrichment (Lower) analysis of both up-regulated and down-regulated DEGs. **E** Flow cytometry validation of HLA-C^high^ PBMCs from CTRL (Upper) and IgAN (Lower). **F** Statistical analysis of HLA-C^high^ PBMCs from CTRL and IgAN. CTRL n = 7; IgAN n = 12; Mean ± SEM; *p < 0.01

To explore the major transcriptomic changes in PBMCs between CTRL and IgAN, we firstly analyzed the differentially expressed (DE) genes between two groups from all cell clusters. Sixteen genes were found to be significantly down-regulated while four genes were significantly up-regulated (Fig. 2A). Intriguingly, the expression levels of human leukocyte antigen (HLA)-C were significantly up-regulated throughout the cell clusters of IgAN (Fig. 2B). HLA-C, one of the MHC class I heavy chain receptors, modulates the function of NK cells during viral infections, autoimmune diseases, and cancers through the killer cell immunoglobulin-like receptors (KIRs) [36–39]. Compared with HLA-A and HLA-B, HLA-C molecules display a lower surface expression level on somatic cells and less allelic variation in the α1 helix at the binding site for NK cell KIRs. An increased expression level of HLA-C may influence NK cell cytotoxicity functions in IgAN. Another up-regulated gene was Krüppel-like factor 4 (KLF4), which is a transcription factor regulating cell proliferation, differentiation, and migration in various cell types. Unlike HLA-C, the expression level of KLF4 was predominantly up-regulated in classical monocytes, non-classical monocytes, B cells, and plasma cells. KLF4 is reported to be an important regulator that controls the monocyte transcriptional network and regulates monocyte differentiation [40–42]. In B cells, however, KLF4 has a growth-suppressive property and maintains B cell quiescence [43–45]. The other two up-regulated genes, TMEM176A and TMEM176B, were found to be up-regulated in classical and non-classical monocytes (Fig. 2B). TMEM176A and TMEM176B have been identified as immunoregulatory endophagosomal cation channels [46–48]. These ubiquitously expressed proteins contain four transmembrane domains and an ITIM motif in its C terminus and suppress inflammasome [49]. Up-regulation of TMEM176A and TMEM176B may modulate monocyte inflammasome in IgAN. In contrary to the up-regulated DE genes, the down-regulated ones were mostly assigned to NK and CD8 + T cell functions, such as PFR1, NKG7, GMZA, GMZB, GMZH, and GNLY (Fig. 2C), suggesting decreased cytotoxicity functions of NK and CD8 + T cells. To further understand the transcriptomic differences between CTRL and IgAN, KEGG Pathway and Gene Ontology (GO) analysis were performed using all DEGs. Indeed, the results of both analysis results suggested that the DEGs were enriched in NK cells mediated cytotoxicity, cytolysis, and cell killing functions (Fig. 2D). To validate these scRNA-seq results, we performed flow cytometry and confirmed the significantly increased number of HLA-C^high^ PBMCs from IgAN compared with that from CTRL (Fig. 2E and F), which validated our scRNA-seq results at the protein level.

### scRNA-seq suggested NK cell exhaustion in IgAN

**Fig. 3:**
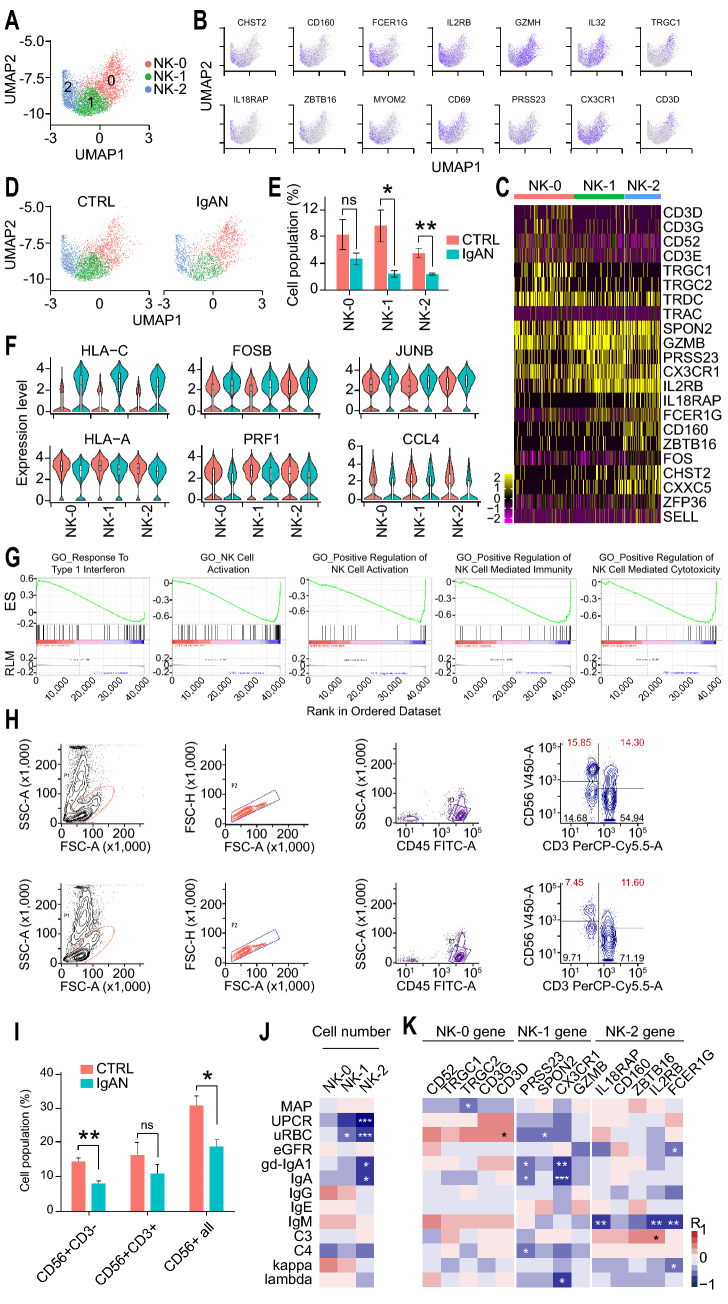
scRNA-seq revealed a decreased number and impaired function of NK cells in IgAN. **A** UMAP illustration of refined NK cell clusters from CTRL and IgAN annotated by their markers genes: NK-0 (Red circles), NK-1 (Green circles), NK-2 (Blue circles). **B** Representative feature plots of the marker genes for NK cell subset annotation. **C** Heatmap of the marker genes contributed from each NK cell subsets. **D** Individual UMAP illustration of NK cells by cells of origin. **E** Statistical analysis of each NK cell subset between CTRL and IgAN. CTRL n = 6; IgAN n = 10; Mean ± SEM; *p < 0.05, **p < 0.01. **F** Violin plots of the DEGs in each NK cell subset between CTRL (Red violin) and IgAN (Blue violin). **G** Enrichment plots from gene set enrichment analysis (GSEA) of GO categories in NK cells between CTRL and IgAN. **H** Flow cytometry validation of the CD56 + CD3 ± NK cells in CTRL and IgAN. **I** Statistical analysis of the cell percentage of CD56 + CD3-, CD56 + CD3 + , and total CD56 + cells between CTRL (Red) and IgAN (Blue). CTRL n = 4; IgAN n = 4; Mean ± SEM; *p < 0.05; **p < 0.01. **J** Heatmap of the Spearman correlation of IgAN clinical parameters with the cell numbers of each NK subset. Red and blue boxes indicate positive and negative correlations, respectively. *p < 0.05; ***p < 0.001. **L** Heatmap of the Spearman correlation of IgAN clinical parameters with the expression levels of marker genes in each NK subset. Red and blue boxes indicate positive and negative correlations, respectively. *p < 0.05; **p < 0.01; ***p < 0.001

Our DEG analysis results of all clusters indicated that both NK cell number and functions might be affected in IgAN (Fig. 1F and 2D). Therefore, we re-analyzed the NK cell cluster alone and divided cells into 3 subsets according to their transcriptomic profiles: NK-0, NK-1, and NK-2 (Fig. 3A and B). Each subset exhibited distinct gene expression patterns (Fig. 3C). NK-0, but not NK-1 or NK-2, expressed high levels of T cell marker genes, including CD3D, CD3G, and CD3E, which indicates that this subset was predominantly NKT cells. NK-1 expressed high levels of CX3C chemokine receptor 1 (CX3CR1) while NK-2 expressed high levels of IL2RB and FCER1G (Fig. 3B and C). Next, we compared the percentage of each NK cell subset between CTRL and IgAN, and found that the numbers of NK-1 and NK-2 were significantly decreased in IgAN (Fig. 3D and E). The up-regulated DEGs in IgAN NK cells were HLA-C, FOSB, and JUNB. FOSB and JUNB are two principal components of the activating protein-1 (AP-1) complex, which has been linked to various cell activities and functions [50]. Deregulated expression of AP-1 subunits might cause harmful immune cell activation during chronic inflammation and autoimmune diseases [51]. Previous reports suggest that FOSB and JUNB regulate cell proliferation through various mechanisms [52, 53]. Up-regulation of HLA-C, together with down-regulation of cytotoxic effector molecule PRF1, and chemokine CCL4, indicated an impaired function of NK cells in IgAN (Fig. 3F). Gene Set Enrichment Analysis (GSEA) identified that these DEG networks were enriched in response to IFN-I, NK cell activation, as well as NK cell-mediated cytotoxicity and immunity (Fig. 3G). These results suggested that NK cell activation and cytotoxicity functions in IgAN were decreased compared with those in CTRL, albeit with high response to IFN-I, which is critical to promote NK cell activation and cytotoxic function during viral infection [54–57]. We, therefore, proposed that NK cells in IgAN could become exhausted. Previous studies have shown that under certain circumstances, such as chronic infections and tumors, NK cells displayed an exhausted status, including poor effector and cytotoxicity functions, which was similar to the exhausted T cells [58–61]. Flow cytometry confirmed the significantly decreased numbers of total NK (CD56 + CD3 ±) as well as NK-1 and NK-2 (CD56 + CD3-) cells in IgAN (Fig. 3H and I). To further understand each NK subset contribution in IgAN, we investigated the correlation of scRNA-seq data and clinical parameters. Intriguingly, we found that the cell numbers of NK subsets were negatively correlated with the clinical parameters of IgAN (Fig. 3J). Lower NK-1 and NK-2 cell numbers were significantly associated with higher levels of UPCR, uRBC, gd-IgA1, and IgA. In addition, NK-1 marker genes PRSS23 and CX3CR1 were negatively correlated with the gd-IgA1 and IgA levels (Fig. 3K). These data suggested that NK cells were exhausted and decreased in IgAN and correlated to disease manifestations.

### scRNA-seq identified DEGs in B cells positively correlated with IgAN.

**Fig. 4:**
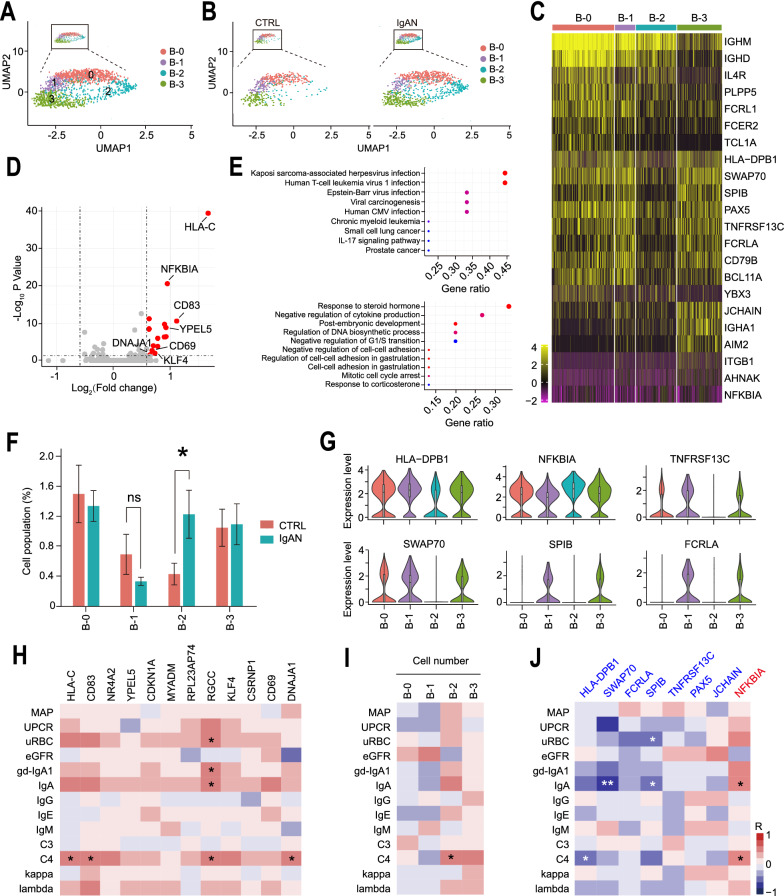
scRNA-seq identified candidate genes and pathways of B cells associated with IgAN. **A** UMAP illustration of refined B cell clusters annotated by their transcriptomic profiles: B-0 (Red circles), B-1 (Purple circles), B-2 (Blue circles), B-3 (Green circles). **B** Individual UMAP illustrations showing the refined B cell clusters from CTRL and IgAN. **C** Heatmap of the marker genes contributed from each B cell subset. **D** Volcano plot showing the up-regulated DEGs in IgAN B cells compared with that in CTRL. **E** KEGG pathway (Upper) and GO enrichment (Lower) analysis of the DE genes in CTRL and IgAN B cells. **F** Statistical analysis of each B cell subset between CTRL and IgAN. CTRL n = 6; IgAN n = 10; Mean ± SEM, *p < 0.05. **G** Representative violin plots of the marker genes in B cell subsets from CTRL and IgAN. **H**–**J** Heatmap of the Spearman correlation of IgAN clinical parameters with the expression levels of DEGs (**H**), the numbers of each B cell subset (**I**), and the marker genes of B-2 subset (**J**). Red and blue boxes indicate positive and negative correlations, respectively. *p < 0.05; **p < 0.01

Decades ago, it has been shown that the releasing of gd-IgA1 from B cells is the critical step, if not the initial, of the “four-hit” hypothesis of IgAN pathogenesis [6, 7, 11]. We, therefore, re-analyzed the B cell cluster and divided them into four subsets: B-0, B-1, B-2, and B-3, according to their transcriptomic profiles (Fig. 4A). Surprisingly, we discovered that the B-2 subset was predominantly found in IgAN (Fig. 4B). Most of these cells expressed conventional B cell marker genes, including CD19 and CD79B. B-0, B-1, and B-2 subsets expressed naïve B cell marker genes, including IGHD, IGHM, and IL4R, while B-3 expressed high levels of JCHAIN and AIM2 (Fig. 4C), which mainly are expressed in memory B cells [62, 63]. We next investigated the DEGs from all B cell subsets between CTRL and IgAN. Interestingly, all the DEGs were up-regulated in IgAN B cells (Fig. 4D). Besides HLA-C, which was up-regulated in all cell clusters, CD83 was the second most up-regulated DEG in IgAN B cells. CD83 is a conserved integral membrane protein, which was originally found to be highly expressed in the peripheral circulating mature dendritic cells. Recent studies indicated that CD83, besides its regulatory role in T cell maturation and activation, is also involved in B cell maturation, proliferation, and function [64–66]. One study has shown that overexpression of CD83 exhibits an inhibitory function in mouse B cells, leading to a reduced level of B cell proliferate, class-switch and secrete immunoglobulin as well as an increased level of the immunoregulatory cytokine IL-10 by marginal zone B cells [64]. However, conditionally deleting CD83 in mouse B cells failed to produce a significant change in response to antigens, albeit some changes were observed in germinal center composition and IgE class-switching [65]. On the contrary, targeting CD83 with a monoclonal antibody in B cells could inhibit their responses to specific antigens in a human PBMC xenograft model without the depletion of pan B cells [66]. CD69, another activation marker of B cells, was also up-regulated in IgAN B cells. Therefore, we believed that B cells may had increased activation and response to specific antigens in IgAN. Intriguingly, NFΚBIA (NFKB inhibitor alpha) was found to be up-regulated, indicating low NFκB activity in IgAN B cells (Fig. 4D). YPEL5 is a highly conserved gene in eukaryotic cells and is involved in certain cell functions, such as cell division [67, 68]. Increased YPEL5 level suggested increased cell proliferation in IgAN B cells. Another interesting DE gene was DNAJA1, which acts as heat shock protein 70 cochaperones, facilitating protein folding, trafficking, prevention of aggregation, and proteolytic degradation. In humans, this gene has been implicated in the positive regulation of the influenza A virus replication through co-option by the virus [69]. Unexpectedly, KEGG and GO analysis showed that these DEGs were mostly enriched in virus infection-related pathways, including Kaposi sarcoma-associated herpesvirus, HTLV, EBV, and CMV (Fig. 4E), which indicated that B cells’ alterations in IgAN were related to virus infection.

### A subset of B cells was closely associated with IgAN

We next compared the cell population of each B cell subset between CTRL and IgAN. Statistical analysis showed a significant increase in the number of B-2 cells of IgAN compared with that of control (Fig. 4F). We also observed fewer B-1 cells in IgAN, although there was no statistical difference between the two groups (Fig. 4F). To further study and understand the B-2 subset, we investigated their marker genes and discovered six marker genes of the B-2 subset, where only one gene, NFΚBIA was up-regulated while the other five were down-regulated (Fig. 4G). NFΚBIA gene encodes IKBα, an inhibitor of the NFκB1 protein, and functions as an inhibitor of NFκB pathway [70]. Increased level of NFΚBIA in the B-2 subset suggested a lower level of NFκB signaling. Another molecule related to NFκB signaling, TNFRSF13C, as known as BAFF receptor (B cell-activating factor receptor, BAFF-R), was down-regulated in B-2 cells. TNFRSF13C, also known as the BAFF receptor, belongs to the TNF receptor superfamily that interacts with BAFF and activates NFκB signaling, which is an essential receptor for B cell maturation and survival [71]. Interestingly, BAFF-R is reported to be down-regulated in B cells of SLE patients, suggesting a regulatory role in B cells under SLE disease conditions [72, 73]. Our scRNA-seq indicated that the expression level of TNFRSF13C was down-regulated in the B-2 subset (Fig. 4G). With the increased level of NFΚBIA and decreased level of TNFRSF13C, we believed that the B-2 subset exhibited an overall decreased level of NFκB signaling activity. Intriguingly, some recent clinical cases showed that anti-TNF alpha therapies (Adalimumab/Infliximab/Certolizumab) may be associated with the pathogenesis of IgAN [74–78]. Although the detailed mechanism was yet to be elucidated, our scRNA-seq results suggested a new working hypothesis for this clinical observation: Anti-TNF-alpha could inhibit NFκB and might disrupt normal B cell functions and contribute to the pathogenesis of IgAN. HLA-DPB1, an HLA class II molecule, plays an essential role in the adaptive immune system by presenting viral peptides in antigen-presenting cells. Down-regulation of HLA-DPB1 in B-2 cells suggested a decreased ability of antigen presentation in response to viral infection. SWAP-70 is a component of an enzyme complex that involves B cell activation signaling through recombination immunoglobulin class switch regions. The other two low-expression genes were FCRLA and SPIB, which both are involved in B lymphocyte development. FCRLA and SPIB also demonstrated decreased expression in the B-0 subset, indicating that the B-2 subset may be closely related to naïve B cells. Overall, these scRNA-seq data indicated that transcriptomic abnormalities in the B-2 subset. We next interrogated the correlation of IgAN clinical parameters with the DEGs. We found the DEGs were mostly associated with IgAN severities, including uRBC, gd-IgA1, IgA, and C4 (Fig. 4H). In addition, we found that B-2 cell number was positively associated with the levels of UPCR, uRBC, gd-IgA1, IgA, and C4, but was negatively associated with eGFR, which strongly indicated that the B-2 subset incresed with the progression of IgAN. Although the B-3 subset was also positively associated with these data, it was positively associated with eGFR, suggesting a complex role of B-3 cells in IgAN. B-0 and B-1 subsets were negatively associated with the levels of gd-IgA1 and IgA, suggesting they may have protective effects in IgAN (Fig. 4I). Finally, we examined the Spearman correlation of IgAN clinical parameters with the high-expressed and low-expressed genes in the B-2 subset (Fig. 4J). Surprisingly, we discovered that all the low-expressed genes were negatively associated with the levels of gd-IgA1 and IgA, whereas the high-expressed gene, NFΚBIA was positively associated with gd-IgA1 and IgA levels. Taken together, these data suggested that a specific B cell subset, which exhibited low NFκB activation level, has a strong association with IgAN and the detail mechanisms required further investigations.

### A classical monocyte subset positively correlated with IgAN clinical parameters.

**Fig. 5:**
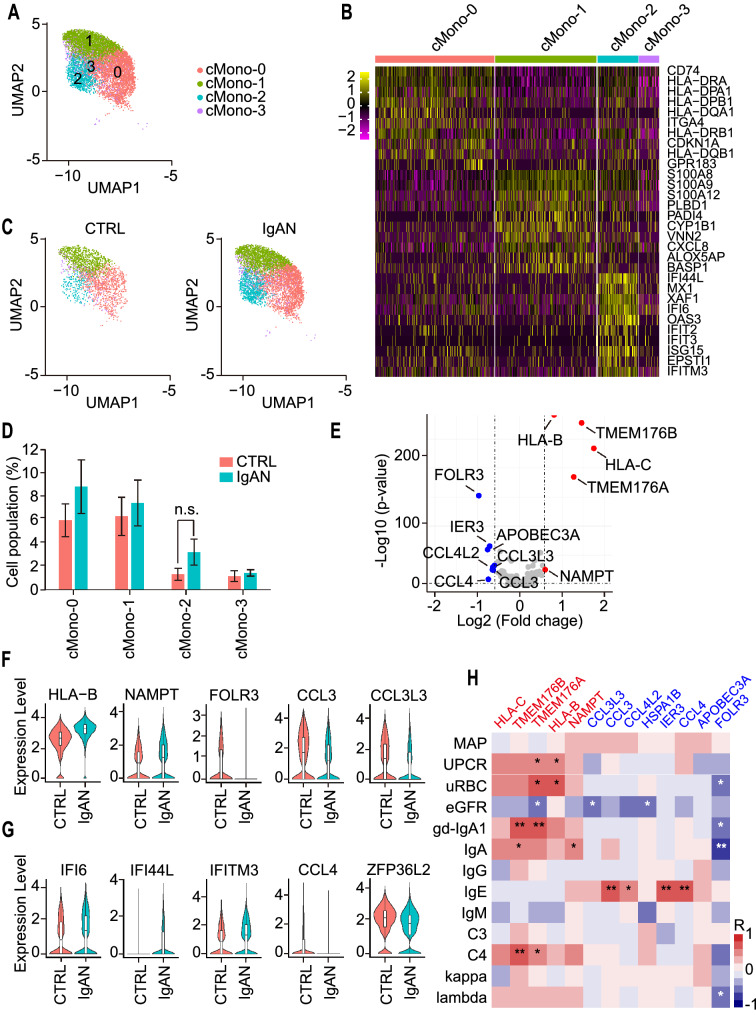
scRNA-seq identified DE genes of classical monocytes closely associated with the clinical parameters of IgAN. **A** UMAP illustration of refined monocyte cell clusters from CTRL and IgAN annotated by their marker genes: cMono-0 (Red circles), cMono-1 (Green circles), cMono-2 (Blue circles), cMono-3 (Purple circles). **B** Heatmap of the marker genes contributed from each classical monocyte subsets. **C** Individual UMAP illustration of classical monocytes by cells of origin. **D** Statistical analysis of each classical monocyte subset between CTRL and IgAN. CTRL n = 6; IgAN n = 10; Mean ± SEM. **E** Volcano plot showing both up- (Red circles) and down-regulated (Blue circles) DE genes in IgAN classical monocytes compared with that in CTRL. **F**, **G** Representative violin plots of newly discovered (**F**) and previously reported (**G**) DEGs in IgAN classical monocytes. **H** Heatmap of the Spearman correlation of IgAN clinical parameters with the expression levels of up- (Red circles) and down-regulated (Blue circles) DEGs in classical monocytes. Red and blue boxes indicate positive and negative correlations, respectively. *p < 0.05; **p < 0.01

Since both a recent IgAN scRNA-seq study and our results indicated that monocytes significantly changed in IgAN [30], we re-analyzed the classical monocyte cluster alone and divided it into 4 subsets according to their transcriptional profiles: cMono-0, cMono-1, cMono-2, and cMono-3. cMono-0, -1, and -2 exhibited distinct cell populations while cMono-3 a diffused pattern (Fig. 5A). The heatmap showed that cMono-0 expressed high levels of MHC-II molecules, cMono-1 expressed high levels of S100A8, S100A9, and chemokine CXCL8, cMono-2 expressed high levels of interferon-induced genes, while cMono-3 expressed low levels of these genes (Fig. 5B). When comparing the percentages of these cell subsets between CTRL and IgAN, we observed a slight increase in cell number of the cMono-2 subset in IgAN, albeit with no statistical significance (Fig. 5C and D). Interestingly, three up-regulated DEGs from classical monocytes, HLA-C, TMEM176A, and TMEM176B, also appeared in the DEGs from all cell clusters, which indicated that the monocytes were the main driving component for these DE genes. Besides HLA-C, HLA-B was also up-regulated, thus it is more convinced that monocytes in IgAN had increased level of MHC class I molecule. We found that NAMPT, a key enzyme in NAD salvage pathway and catalyzes the generation of NAD, increased in classical monocytes of IgAN too. NAMPT was increased in several inflammatory conditions including rheumatoid arthritis, inflammatory bowel disease and psoriasis; moreover, inhibition of NAMPT is beneficial in some preclinical animal models [79]. The down-regulated DE genes were mostly chemokines, including CCL3, CCL4, CCL3L3, and CCL4L2, which indicated that monocyte-mediated chemotaxis was impaired in IgAN (Fig. 5E and F). A recent study suggested that the interferon pathway in classical monocytes was modified in IgAN, with increased levels of IFI6, IFI44L, and IFITM3, and decreased levels of CCL4 and ZFP36L2 [30]. We then looked through our scRNA-seq results and confirmed a similar tendency in these genes (Fig. 5G). Next, we performed the Spearman correlation analysis of IgAN clinical parameters with the expression levels of up- (Red) and down-regulated (Blue) DEGs. Surprisingly, we discovered that all of the up-regulated DE genes (HLA-B, HLA-C, TMEM176A, TMEM176B, and NAMPT) in classical monocytes were positively correlated with IgAN clinical parameters, including UPCR, uRBC, gd-IgA1, IgA, and C4. Whereas, one of the down-regulated DEG, Folate Receptor Gamma (FOLR3), showed a strong negative correlation with these parameters (Fig. 5H). In summary, the transcriptomes of classical monocytes in IgAN were substantially altered with increased levels of MHC class I molecules and TMEM176A/B, and decreased level of FORL3. It is of considerable interest to explore how these alterations contribute to the development and progression of IgAN.

### scRNA-seq revealed the dynamics of the intercellular communication networks in IgAN

**Fig. 6:**
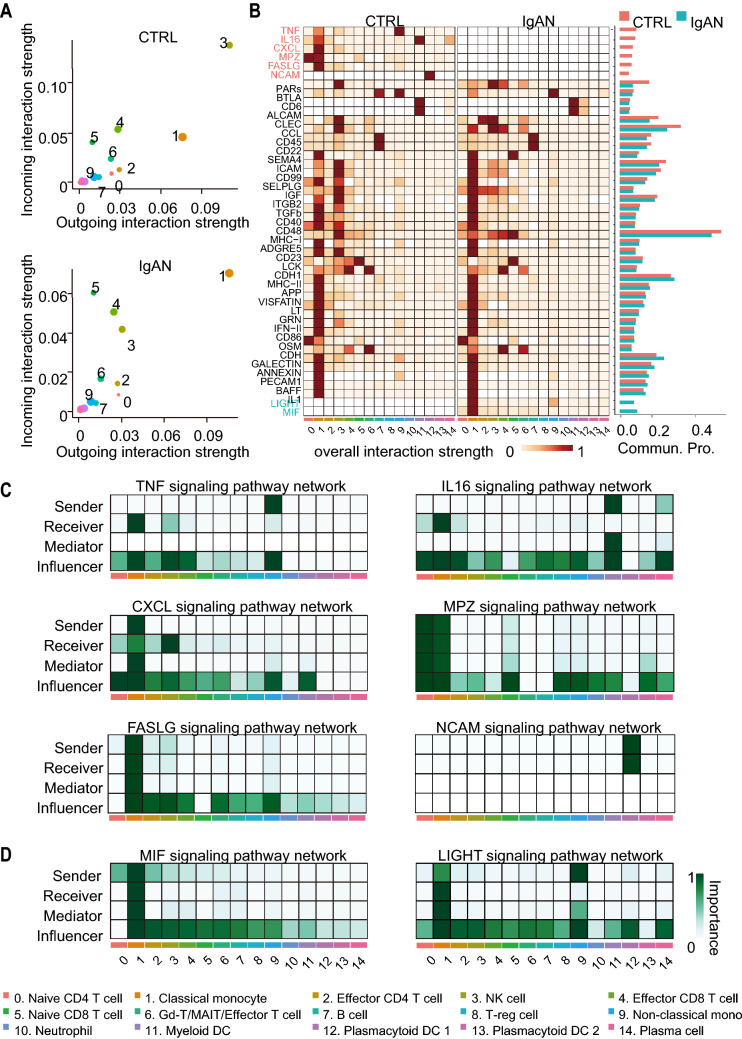
CellChat analysis of the intercellular communication networks in PBMCs from CTRL and IgAN. **A** Scatter plots of the incoming and outgoing interaction strengths in each cluster of CTRL (Upper) and IgAN (Lower). **B** Heatmap shows the relative communication strength of the inferred signaling pathways in each cluster of CTRL and IgAN. The bar graph shows the overall information flow in each cluster of CTRL and IgAN. The signaling pathways colored by red and blue are enriched in CTRL and IgAN, respectively. **C**, **D** Relative contribution heatmaps of the dominant sender, receiver, mediator, and influencer in the inferred signaling pathways enriched in CTRL (**C**) and IgAN (**D**), respectively

Intercellular communications and interactions of immune cells orchestrate host immunity. Increasing evidence suggests that changes in the intercellular communication networks are strongly associated with disease pathogenesis. To understand the network in IgAN, we investigated the cell–cell communication strength and probability (i.e., Information flow) in PBMCs from CTRL and IgAN using CellChat, a tool for exploring the cell–cell communication network [80]. Firstly, we compared the outgoing and incoming interaction strength to identify cell populations with significant changes in sending or receiving signals between CTRL and IgAN. We observed that NK cells (Cluster 3) and classical monocytes (Cluster 1) emerged as the major affected sources and targets in IgAN compared with that in CTRL. NK cells exhibited reduced levels in outgoing and incoming interaction strength whereas monocytes showed increased levels in both (Fig. 6A). We subsequently compared the overall relative strength of the identified signaling pathways in each cell cluster by its aggregated outgoing and incoming signaling. Significant signaling pathways were ranked based on the differences in the overall communication probability in the indicated networks between CTRL and IgAN (Fig. 6B). Five signaling pathways (TNF, IL16, CXCL, MPZ, FASLG, and NCAM) were found to be unique in CTRL, while two (MIF, LIGHT) were unique in IgAN. We next explored the contributors of these seven signaling pathways by identification of the dominant senders, receivers, mediators, and influencers in the inferred network by computing several network centrality measures for each cell cluster. Interestingly, we found that classical monocytes were the key player in these signaling pathways. In CTRL, classical monocytes were the major receiver of TNF, IL16, MPZ, FASLG, and the major sender of CXCL to NK cells (Fig. 6C) and it became the major receiver of MIF and LIGHT in IgAN (Fig. 6D). Surprisingly, the previously reported intercellular communication signaling pathways between NK cells and monocytes were found to be largely modified in IgAN. For example, IFN-II and SELPLG, which were predominantly secreted from NK cells in CTRL, were found to be secreted from classical monocytes in IgAN, allowing classical monocytes to become self-regulated (Additional file 1: Fig. S6A). This result could partially explain why classical monocytes showed increased interferon signaling even with a suppressed function of NK cells in IgAN. In addition, the receiver of ICAM and TGFb pathways was changed from NK cells to monocytes (Additional file 1: Fig. S6A). Previous studies suggested that BAFF and IL1 signaling pathways played important roles in IgAN pathogenesis, therefore we explored their networks in our dataset. However, neither the interaction strength nor the communication probability was significantly affected in IgAN compared with that in CTRL (Additional file 1: Fig. S6B), which might be due to the insufficient sequencing depth. Taken together, these results strongly indicated that the intercellular communication networks between NK cells and monocytes were disrupted.

Over the past decades, GWAS greatly advanced our knowledge in the genetic architecture and susceptibility in IgAN pathogenesis and provided novel insights into the understanding of IgA nephropathy [16–18]. The MHC locus on chromosome 6p21 is strongly linked to immune-related glomerulopathies, with a distinct association pattern between HLA alleles and different types of renal diseases, suggesting various immune-related mechanisms in these diseases. Therefore, we integrated previously reported GWAS genes with our scRNA-seq data and examined the expression levels of each cell cluster from CTRL and IgAN (Additional file 1: Fig. S7). We found that MHC class I molecule HLA-A was expressed in all cell clusters and exhibited decreased levels in NK cells, effector CD8 T cells, Tregs, plasmacytoid DC1, and plasma cells from IgAN. Whereas the MHC class II molecules were not affected between CTRL and IgAN. The expression level of TAP1, which participates in the antigen-presenting process, was increased in neutrophils and decreased in plasma cells in IgAN. PSMB8 and PSMB9 were both increased in plasmacytoid DC1. ITGAX was found to be decreased in myeloid DC in IgAN. We did not observe a strong expression of CFH, CFHR1, CFHR2, CFHR3, CFHR4, CFHR5, VAV3, CARD9, ACCS, EXT2, TNFSF13, MPDU1, HORMAD2, LIF, and OSM, which may be attributed to either low expression levels of these genes in immune cells or the sequencing depth limitation in this study. Nonetheless, our scRNA-seq data offered directions and clues on studying the immune cell-related GWAS genes. Further studies need to be performed to investigate the detailed mechanisms of these genes in the pathogenesis of IgAN.

## Discussion

Considerable evidence implicates that mesangial immune-complex deposits are originated from the circulating immune complexes in IgAN. First of all, serum gd-IgA1-IgG immune complexes are increased in most IgAN patients [6, 12, 81, 82]. Secondly, IgAN recurs in 30–50% of patients after kidney transplantation [83–85]. In addition, a study reported that immune complex deposits disappeared within weeks in a kidney with subclinical IgAN after transplanted to a patient without IgAN [86]. Therefore, circulating gd-IgA1-IgG immune complexes possibly play critical roles in the pathogenesis of IgAN, while kidneys may be “innocent bystanders” [87], although some studies indicate that mesangial cells express receptors prone to immune complexes deposition. To date, however, neither the identity of the gd-IgA1 secreting cells nor the gd-IgA1 production mechanism is clear. Some evidence indicates that gd-IgA1 producing cells are located in the respiratory and gastrointestinal mucosal tissues, while some studies found that IgA-producing cells in the bone marrow, which may secrete small amount of polymeric IgA1 after infection. Interestingly, an in vitro study investigated the enzymatic activity in IgA1-producing cells using peripheral blood cells and observed a decreased activity level in β-1,3-galactosyltransferase, and an increased level in N-acetyl-galactosamine-α-2,6-sialyltransferase [88], suggesting that PBMCs may contribute to IgAN pathogenesis.

In this study, we performed scRNA-seq and analyzed PBMCs from 6 healthy donors and 10 IgAN patients. Our scRNA-seq results, together with flow cytometry validation data, indicate three major immune cell types, including natural killer cells, B cells, and monocytes are closely associated with IgAN. Human NK cells bind to their major ligands HLA class I molecules with a group of surface receptors, killer cell immunoglobulin-like receptors (KIRs) [89]. NK cell tolerance toward normal cells relies on the expression of inhibitory receptors binds to HLA class I molecules, such as KIR, NKG2A/CD94, and LIR1. At disease conditions, NK cells attack and lyse the abnormal cells with the decreased surface expression level of HLA-I molecules, which is also known as the “missing self” [90]. Therefore, the presentation of host peptides by HLA-I molecules allows self-recognition and immune tolerance, as well as NK cell licensing. In virally infected cells and certain cancer cells, loss of surface expression of HLA-I results in avoiding CD8 + T cell attacking. As a consequence, these HLA-I deficient cells can be attacked and eliminated by NK cells rather than CD8 + T cells. Among HLA-I molecules, HLA-C is closely homologous to HLA-A and -B, but it is unique on several things: Firstly, HLA-C contains a conserved KYRV motif in α1 domain, a conserved glycine (Gly) at amino acid 45 [91], and four conserved sequences in the α2 domain. Different from HLA-A and HLA-B, the peptide binding region of HLA-C displays a reduced level of diversity, which is highly selective to self-peptides [92, 93]. Finally, HLA-C has a low expression level compared with HLA-A and -B. It is found to be expressed at only 10% cells, which is 15–35% less than the other two HLA molecules [94]. Because of its reduced diversity in the peptide-binding region, studies have shown that HLA-C binds more specifically to NK cells, while HLA-A and -B bind more specifically to cytotoxic T cells [95]. Here we found a ubiquitous up-regulation of HLA-C expression level in all cell types, suggesting a strong inhibition of NK cell activation and cytotoxicity functions in IgAN. Currently, the mechanism of HLA-C up-regulation in IgAN is not clear, but several studies indicate that chronic viral infection can up-regulate HLA-C expression level and inhibit NK cell cytotoxicity function [96, 97]. GSEA analysis results confirm that NK cell activation and cytotoxicity indeed are reduced in IgAN, although they exhibit a high level of type 1 interferon response. Thus, we purpose that NK cells became exhausted in IgAN probably due to chronic infection and/or inflammation. Since NK cell number and marker genes are negatively correlated with IgAN clinical parameters, the relationship of NK cell exhaustion with disease progression and prognosis in IgAN merits further investigation.

Evidence from both in vitro and in vivo models implicates that B cells play a critical role in IgAN pathogenesis. Intriguingly, transfusion of spleen cells without pan T cells and transfusion of CD19 + B cells to SCID mice reconstitutes human IgAN phenotype in mouse, which indicates that the gd-IgA1-producing B cells in IgAN function through a T-cell-independent manner [98]. In this study, we have unveiled a novel B cell subset, which is predominantly found in IgAN patients. The B-2 cell number and genes are closely associated with IgAN data, including gd-IgA1 and IgA levels. Interestingly, the expression level of TNFRSF13C (BAFF-R) is down-regulated in B-2 cells. In B cells, BAFF-R is expressed from the immature stage until the generation of plasma cells. Bacterial polysaccharides and HIV have been shown to reduce the BAFF-R expression in B cells. A decreased expression level of BAFF-R has also been observed in the *P. falciparum*-infected children as well as adults, although the detailed mechanism is unelucidated. *T. brucei*-infected mice also downregulated the BAFF-R expression level in marginal zone B cells, which leads to a decreased number of anti-parasite B cells. Interestingly, a clinical cohort study proposed that *Streptococcus pyogenes* infection was strongly associated with the BAFF production in IgAN patients, however, a decreased level of BAFF was observed [99]. Activation of BAFF signaling in vitro by treatment tonsillar mononuclear cells of IgAN patients could induce secretion of gd-IgA1 through reducing the expression level of C1GALT1 [100, 101]. Furthermore, increased levels of BAFF and TLR9 are correlated to the serum level of IgA1, renal function, and disease progression [102]. However, a study of BAFF-overexpressing Tg mouse models and clinical parameters indicated that a specific elevation of APRIL but not BAFF levels in peripheral blood from IgAN patients, although BAFF Tg mice indeed developed IgAN [21]. Hence Martín-Penagos et al. raised a hypothesis that this phenomenon is due to the up-regulation of APRIL-induced responses and down-regulation of BAFF-induced responses in B cells of IgAN patients. Compared with BAFF, APRIL is more strongly correlated to IgA class switching upon binding to TACI [103].

Our scRNA-seq results also show that B-2 cells exhibit an increased level of NFΚBIA, an inhibitory molecule for NFκB pathways, together with a decreased level of BAFF-R, strongly suggesting NFκB pathways are reduced in B-2 cells. Because B-2 cells have a distinctive expression pattern and they are closely related to the IgAN, they have great potential to become a novel biomarker and target in IgAN. Yet further study using cell and animal models need to be done to examine the detailed molecular mechanisms of B-2 cells in IgAN pathogenesis.

Monocytes play a crucial role in immune response and may contribute to the pathogenesis of IgAN. One study has shown that monocytes from IgAN patients display a higher respiratory burst compared with those from healthy controls [35]. A study has shown that metabolic activation of monocytes linked to oxidative stress, chronic inflammation, and kidney disease progression. Short-term and low-dose treatment monocytes with atorvastatin, a selective, competitive inhibitor of HMG-CoA reductase, reduce the monocyte respiratory burst [35]. Another study uncovers that the apoptotic pathway and mitochondrial function genes are altered in non-classical monocyte in IgAN [33]. In particular, the elevated expression of NDUFS3 and TNFRSF1A suggested the altered mitochondrial respiratory function and death receptor homeostasis in monocytes. Intriguingly, two recent studies have confirmed that TNF gene expression levels in monocytes from IgAN patients are reduced compared with those from healthy donors [30, 34], further suggesting a reduced TNF alpha signaling pathway in IgAN patients. In our scRNA-seq results, we find a classical monocyte subset is slightly increased in IgAN. Differentially expressed gene analysis reveals that IgAN monocytes up-regulate two MHC class I molecules, HLA-B and HLA-C, which inhibit cytotoxic T cells and NK cells, respectively. The other two up-regulated genes are TMEM176A and TMEM176B, which regulate DC maturation and ablates inflammasome-derived caspase-1/IL1β [48, 104, 105]. Up-regulation of these genes indicates impaired monocyte maturation and IL1β signaling pathway in IgAN. Impaired monocyte function may subsequently affect other immune cells through TNF alpha and NFκB signaling pathways. Analysis of intercellular communications strongly suggested that the interaction networks in NK and classical monocytes were modified in IgAN, where the classical monocytes became auto-stimulated and self-regulated in IgAN.

Over the past decades, GWAS greatly advanced our knowledge in the genetic architecture and susceptibility in IgAN pathogenesis and provided novel insights into the understanding of IgA nephropathy. To date, approximately 20 genome-wide significant loci with small to moderate effects have been discovered in IgAN, highlighting its complex polygenic architecture. Many loci, including MHC class I and class II loci, have been shown to function in multiple types of immune cells, thus bringing difficulties to explain the possible contribution mechanism of these genes in IgAN pathogenesis. By integration with our scRNA-seq results, we have been able to clarify the expression levels of previously reported GWAS genes in each immune cell type, which offers new ways to understand the involvement of GWAS genes in IgAN.

The study reported herein has several limitations. First, though this study included as many as 16 participants, these participants are all from a single center, which may introduce selection bias. The results should be tested in a multi-centric study. Second, our present study discovered some interesting and novel changes of NK, B cells, and monocytes in IgAN while further experiments, including in vitro or in vivo functional validation experiments, are required to address the underlying mechanisms.

## Conclusions

We provide a single-cell landscape and resource of immune cells in IgAN, highlighting the remarkable alterations of NK, B cells and monocyte. Our results will facilitate the development of novel biomarkers, diagnostic techniques, and targeted therapies to IgAN as well as other glomerulonephritis diseases.

## Methods

### Clinical characteristics of healthy control donors and IgAN patients

We studied a total number of 19 patients with newly-diagnosed biopsy-proven primary IgAN and 11 healthy control donors. 10 IgAN patients and 6 healthy control donors were used for scRNA-seq and the rest were included in the validation experiments. All of the patients were diagnosed within 6 months. All of the patients displayed mesangial IgA deposition, hematuria, proteinuria, low-level serum albumin, and typical morphologic alterations detected by light and electron microscopy (Additional file 1: Table S1 and S2). None of the patients were diagnosed with Henoch-Schonlein purpura. None of the patients and control donors have received corticosteroid treatment or immunosuppressive therapy before entry into the study. None of the patients or control donors had clinical infectious symptoms at the time point when the study samples were taken. Written informed consents were obtained from all healthy control donors and IgAN patients. Experimental and research procedures were approved by and in accordance with the internal review board and human subject guidelines of the Sun Yat-sen Memorial Hospital and Sun Yat-sen University.

### Peripheral blood mononuclear cell preparation and cryopreservation

Whole blood samples (5 mL) were collected from healthy control donors and IgAN patients. Blood samples were incubated with Red Blood Cell Lysis Buffer (C3702, Beyotime, Shanghai, China) for 5 min and then centrifuged for 5 min at 1000 r.p.m. RT. PBMCs were washed three times with 2 mL PBS. About 10^7^ cells were resuspended in 1 mL freezing media (90% heat-inactivated Fetal Bovine Serum and 10% DMSO) and transfer the cryovials into a gradient freezing container Mr. Frosty™ and put into a -80 °C freezer. After approximately 16 h, the cryovials were transferred into liquid nitrogen until the days of usage.

### Flow cytometry

Whole blood (100 μL) was incubated with Red Blood Cell Lysis Buffer (C3702, Beyotime, Shanghai, China) for 5 min and then centrifuged for 5 min at 1000 r.p.m. RT. After washing with 2 mL PBS three times. PBMCs were stained 30 min with the following antibodies: CD45-FITC (555482; BD Biosciences), CD3-PerCP-Cy5.5 (560835; BD Biosciences), CD4-PE (555347; BD Biosciences), CD8-PE (555367; BD Biosciences), CD19-APC-H7 (560177; BD Biosciences), CD56-PE-Cy7 (557747; BD Biosciences) and CD14-APC (555399; BD Biosciences). and Flow cytometry panels were designed as follow: Panel one for CD45, CD3, CD14, CD19, CD4, and CD56; Panel two for CD45, CD3 and CD8. After incubation, PBMCs were washed three times and resuspended with 200 μl PBS. Flow cytometry experiments were performed by FACSVerse flow cytometer using FACSSuite software (BD Biosciences, San Jose, USA).

### Fluorescence-activated cell sorting (FACS)

Cryopreserved PBMCs were resuscitated, and stained with CD45-FITC antibody and live/dead cell dye Propidium Iodide (556463; BD Biosciences, San Jose, USA). PBMCs were sorted by FACS Jazz flow cytometer (BD Biosciences, San Jose, USA) using BD FACS software (BD Biosciences, San Jose, USA). Sorted PBMCs were used for scRNA-seq with the BD Rhapsody platform.

### scRNA-seq with BD Rhapsody platform

Sorted peripheral blood mononuclear cells were resuspended in PBS with 10% FBS and then centrifuged at 1000 r.p.m. for 5 min and resuspend in 200 μL staining buffer (BD Biosciences, San Jose, USA). Cells from each person were incubated with 10 μL one BD SMK sample tag (633781, BD Biosciences, San Jose, USA) for 20 min at RT and washed with cold-PBS twice. After the sample tag labeling, PBMCs from four people (at least one from healthy donors) were mixed for one scRNA-seq experiment. The average cell number of scRNA-seq experiments was around 15,000 cells. The read depth of each experiment was sequenced to 100 K reads per cell. Single-cell mRNA capture and library construction were performed according to BD Rhapsody's official instructions. Next-generation sequencing was performed using the Illumina Nova-Seq platform according to the official instructions.

### BD Resolve analysis pipeline

Raw reads were processed according to the Whole-Transcriptome Assay Analysis Pipeline (WTA pipeline) of the BD Rhapsody platform. In short, low sequencing quality read pairs were discarded under the following filtering criteria: R1 and R2 reads less than 66 and 64, respectively; the mean base quality score of either reads less than 20; single nucleotide frequency (SNF) higher than 0.55 and 0.80 for R1 and R2 reads, respectively. The filtered R1 reads are used to identify the cell label section sequence (CLS), common sequences (L), Unique Molecular Identifier (UMI) sequence, and poly(T) tail, and the filtered R2 reads were mapped to the reference genomic sequences using Bowtie2 version 2.2.9. To remove the effect of UMI errors on molecule counting derived from PCR error, sequencing bias, and library preparation steps, algorithms of recursive substitution error correction (RSEC) and distribution-based error correction (DBEC) developed by BD were used. Finally, the combination of putative cell data with RSEC/DBEC-corrected molecule results was used to generate the single-cell expression matrix.

### Downstream analysis with Seurat V3.0

The R packages Seurat V3.0 were used to analyze the matrix, integrate and normalize datasets, perform dimensionality reduction, clustering, and differential expression genes. For integrated analysis of our scRNA-seq datasets, we applied canonical correlation analysis (CCA) of the Seurat alignment method for four scRNA-seq datasets [106]. Cell clustering was performed using high variable genes, and the principal components based on high variable genes were used to generate the graphs at a resolution of 0.4.

### GO and KEGG enrichment analysis

The cluster Profiler R package was used to performed Gene Ontology (GO) enrichment analysis of DEGs, in which gene length bias was corrected. GO terms with corrected P value less than 0.05 were considered significantly enriched by DEGs. Cluster Profiler R package was used to test the statistical enrichment of DEGs in KEGG pathways.

### CellChat analysis

Intercellular communication networks were analyzed using Cellchat, which has been built to quantitively infer and analyze cell–cell communications of scRNA-seq data [80]. Briefly, we used assigned cell labels as input and modeled the communication probability, and identified the significant communication networks. For this study, we used the default setting with Identify over-expressed genes Threshold PC = 0.1, Communication probability population size = TRUE. The scRNA-seq data were further visualized as outputs for different analytical tasks through approaches from graph theory, pattern recognition, and manifold learning.

### Statistics

Statistical analysis was performed with SPSS 16.0 and GraphPad Prism 6.0. Student t-test and Mann–Whitney U-test were used to examine the statistical difference. To examine the relationships between two variables, Spearman’s correlation analysis was performed. All data were presented as Mean ± SEM unless otherwise described.

## Supplementary Information

**Additional file 1:** **Table S1.** Clinical characteristic of healthy control subjects and IgAN patients for scRNA-seq. **Table S2.** Oxford classification of IgAN patients for scRNA-seq. **Figure S1.** FACS for PBMCs and scRNA-seq QC results. (A) Representative FACS data of CD45+ PBMCs for scRNA-seq. (B) Distribution plots of gene count to cell number from four scRNA-seq experiments. Each scRNA-seq experiment has four PBMC samples with at last one sample from CTRL. (C, D, E) Summarized results of the RNA counts (C), RNA feature numbers (D), and mitochondria RNA percentages (E) of four scRNA-seq. (F) Correlation between RNA counts and RNA feature number from individual samples. (G) Bar graphs of cell numbers from each sample before and after QC. (H) Summary of cell numbers before and after QC in CTRL, IgAN, and all samples. (I) Pie chart of cell percentages from each sample. **Figure S2.** Data integration of four scRNA-seq results with Seurat V3.0. (A) UMAP illustration of PBMCs from four scRNA-seq results before and after data integration colored by experimental batches. (B) Individual UMAP illustration of PBMCs after data integration colored by cell-type annotation. **Figure S3.** UMAP illustrations of the representative marker genes used for cell type annotation. CD3D, CD4, IL6SThigh for cluster-0 Naive CD4 T cells; CD14 for cluster-1 classical monocytes: CD3D, CD4, IL6STlow for cluster-2 Effector CD4 T cells; NCAM1, FCGR3A for cluster-3 natural killer cells; CD8A, CD8B, IL6STlow for cluster-4 Effector CD8 T cells; CD8A, CD8B, IL6SThigh for cluster-5 Naïve CD8 T cells; KLRB1, KLRG1, GMZK, TRDC, CD8A for cluster-6 gd-T/MAIT/Effector T cells. CD19, IGHD, IGHM for cluster-7 B cells; Foxp3 for cluster-8 Treg cells; FCGR3A+, CD14- for cluster-9 non-classical monocytes; XFA1, MX1 for cluster-10 neutrophils; FCER1A, HLA-DRA, HLA-DPA1 for cluster-11 Myeloid DC; STMN1, MKI67 for cluster-12 Plasmacytoid DC1; IRF8, ITM2C for cluster-13 Plasmacytoid DC2; JCHAIN, CD38 for cluster-14 plasma cells. **Figure S4.** Dot plot for the marker gene expression levels and percentages in cell clusters. **Figure S5.** UMAP and cell population bar graphs of each sample from CTRL and IgAN. (A) UMAP illustrations of each PBMC sample colored by cell type. (B) Stacked bar graphs showing the cell-type composition in each PBMC sample. Colors indicate different cell types. CTRL1-6: healthy donors; 61P-63P, 65P-67P, 69P-72P: IgAN patients. **Figure S6.** Heatmaps of the relative communication strength of the inferred signaling pathways in NK and classical monocytes of CTRL and IgAN. (A) Comparison of signaling pathways between NK and classical monocytes in CTRL (Left) and IgAN (Right). (B) Comparison of BAFF and IL1 pathways between CTRL (Left) and IgAN (Right). **Figure S7.** Integration of scRNA-seq with GWAS genes in immune cells from CTRL and IgAN. The expression levels of GWAS genes were shown in each cell cluster of CTRL and IgAN.

## Data Availability

All single-cell RNA sequencing data were deposited in the BIG Submission Portal (https://bigd.big.ac.cn/gsub/) and the Dataset ID is HRA000831.

## Abbreviations

IgAN: IgA nephropathy
CTRL: Control
DEG: Differentially expressed gene
gd-IgA1: Galactose-deficient IgA1
