# Supplementary material for: Single-cell RNA-sequencing reveals distinct immune cell subsets and signaling pathways in IgA nephropathy
Source: Cell Biosci. 2021 Dec 11;11:203. doi: 10.1186/s13578-021-00706-1 (PMC8665497; doi:10.1186/s13578-021-00706-1)
Supplement: Supplementary file 1 — Additional file 1: Table S1. Clinical characteristic of healthy control subjects and IgAN patients for scRNA-seq. Table S2. Oxford classification of IgAN patients for scRNA-seq. Figure S1. FACS for PBMCs and scRNA-seq QC results. (A) Representative FACS data of CD45+ PBMCs for scRNA-seq. (B) Distribution plots of gene count to cell number from four scRNA-seq experiments. Each scRNA-seq experiment has four PBMC samples with at last one sample from CTRL. (C, D, E) Summarized results of the RNA counts (C), RNA feature numbers (D), and mitochondria RNA percentages (E) of four scRNA-seq. (F) Correlation between RNA counts and RNA feature number from individual samples. (G) Bar graphs of cell numbers from each sample before and after QC. (H) Summary of cell numbers before and after QC in CTRL, IgAN, and all samples. (I) Pie chart of cell percentages from each sample. Figure S2. Data integration of four scRNA-seq results with Seurat V3.0. (A) UMAP illustration of PBMCs from four scRNA-seq results before and after data integration colored by experimental batches. (B) Individual UMAP illustration of PBMCs after data integration colored by cell-type annotation. Figure S3. UMAP illustrations of the representative marker genes used for cell type annotation. CD3D, CD4, IL6SThigh for cluster-0 Naive CD4 T cells; CD14 for cluster-1 classical monocytes: CD3D, CD4, IL6STlow for cluster-2 Effector CD4 T cells; NCAM1, FCGR3A for cluster-3 natural killer cells; CD8A, CD8B, IL6STlow for cluster-4 Effector CD8 T cells; CD8A, CD8B, IL6SThigh for cluster-5 Naïve CD8 T cells; KLRB1, KLRG1, GMZK, TRDC, CD8A for cluster-6 gd-T/MAIT/Effector T cells. CD19, IGHD, IGHM for cluster-7 B cells; Foxp3 for cluster-8 Treg cells; FCGR3A+, CD14- for cluster-9 non-classical monocytes; XFA1, MX1 for cluster-10 neutrophils; FCER1A, HLA-DRA, HLA-DPA1 for cluster-11 Myeloid DC; STMN1, MKI67 for cluster-12 Plasmacytoid DC1; IRF8, ITM2C for cluster-13 Plasmacytoid DC2; JCHAIN, CD38 for cluster-14 plasma cells. [file 13578_2021_706_MOESM1_ESM.pdf]

Table.1 Clinical characteristic of healthy control subjects and IgA nephropathy patients for scRNA-seq

	Control (Mean ± SEM)	IgAN (Mean ± SEM)	P value
N	6	10	NA
Male sex, n (%)	3 (50%)	3 (30%)	0.6066
Age (yr)	30.17 ± 1.54	32.50 ± 2.38	0.4947
BMI	23.03 ± 1.41	21.01 ± 0.88	0.4477
Systolic BP (mmHg)	108.33 ± 7.36	114.90 ± 4.20	0.4154
Diastolic BP (mmHg)	74.00 ± 3.53	70.20 ± 2.83	0.4195
Mean arterial Pressure (mmHg)	85.45 ± 4.10	85.10 ± 3.04	0.9465
eGFR (ml/min per 1.73 m^2)	102.17 ± 3.34	103.60 ± 9.70	0.8918
UPCR (mg/g)	57.39 ± 7.60	476.03 ± 106.18	0.0011
Urine RBC（/HPF）	0.25 ± 0.11	53.23 ± 16.30	0.0011
Hemoglobin (g/L)	134.33 ± 4.53	129.00 ± 5.09	0.4883
Total Protein (g/L)	73.33 ± 1.66	68.56 ± 2.77	0.2342
Serum albumin (g/L)	43.43 ± 0.84	35.76 ± 1.29	0.0008
Serum creatinine (µmol/L)	78.67 ± 5.62	80.10 ± 9.25	0.4792
Blood urea nitrogen (mmol/L)	4.58 ± 0.66	5.04 ± 0.37	0.5222
Blood uric acid (µmol/L)	389.17 ± 52.12	334.80 ± 21.06	0.2773
Cholesterol (mmol/L)	5.24 ± 0.49	4.85 ± 0.23	0.4204
Triglyceride (mmol/L)	0.94 ± 0.06	1.17 ± 0.25	0.7860
HDL (mmol/L)	1.26 ± 0.07	1.24 ± 0.11	0.9087
LDL (mmol/L)	3.31 ± 0.31	2.90 ± 0.14	0.1936
Calcium (mmol/L)	2.34 ± 0.03	2.32 ± 0.03	0.6002
Phosphorus (mmol/L)	1.17 ± 0.03	1.20 ± 0.03	0.4842
Fast blood glucose (mmol/L)	5.13 ± 0.11	4.36 ± 0.08	0.0001
ALT (U/L)	15.67 ± 4.76	14.50 ± 1.30	0.7737
AST (U/L)	18.17 ± 1.70	19.20 ± 0.77	0.5377
Serum gd-IgA1 (ng/mL)	2372.80 ± 329.71	5876.48 ± 972.85	0.0034
Serum IgA (g/L)	1.81 ± 0.19	3.30 ± 0.34	0.0020
Serum IgG (g/L)	11.90 ± 0.48	11.91 ± 0.73	0.9962
Serum IgM (g/L)	1.12 ± 0.22	1.09 ± 0.14	0.8978
C3 (mg/L)	1020.83 ± 88.83	992.00 ± 26.00	0.7662
C4 (mg/L)	197.33 ± 19.66	264.80 ± 33.97	0.1723
Kappa (g/L)	2.86 ± 0.17	3.06 ± 0.19	0.4878
Lambda (g/L)	1.43 ± 0.09	1.81 ± 0.15	0.0833

UPCR: urine protein creatinine ratio; Urine RBC: urine red blood cell; HDL: high-density lipoprotein; LDL: low-density lipoprotein; ALT: Alanine Aminotransferase; AST: aspartate aminotransferase.

Table. S2 Oxford classification of IgAN patients for scRNA-seq

Patient ID	Oxford classification system					Mesangial expansion (0~4)*
	M	E	S	T	C	
61P	1	0	1	0	1	2
62P	1	0	0	0	1	2
63P	1	0	1	2	1	2
65P	1	0	0	0	1	2
66P	1	1	0	0	2	2
67P	1	0	0	0	0	1
69P	1	0	1	0	1	2
70P	1	0	0	0	0	1
71P	1	0	1	0	1	2
72P	1	1	1	0	1	3

*Mesangial expansion level: 0. No expansion; 1. Slight; 2. Slight-medium; 3. Medium; 4. Severe

Continued in the next page;

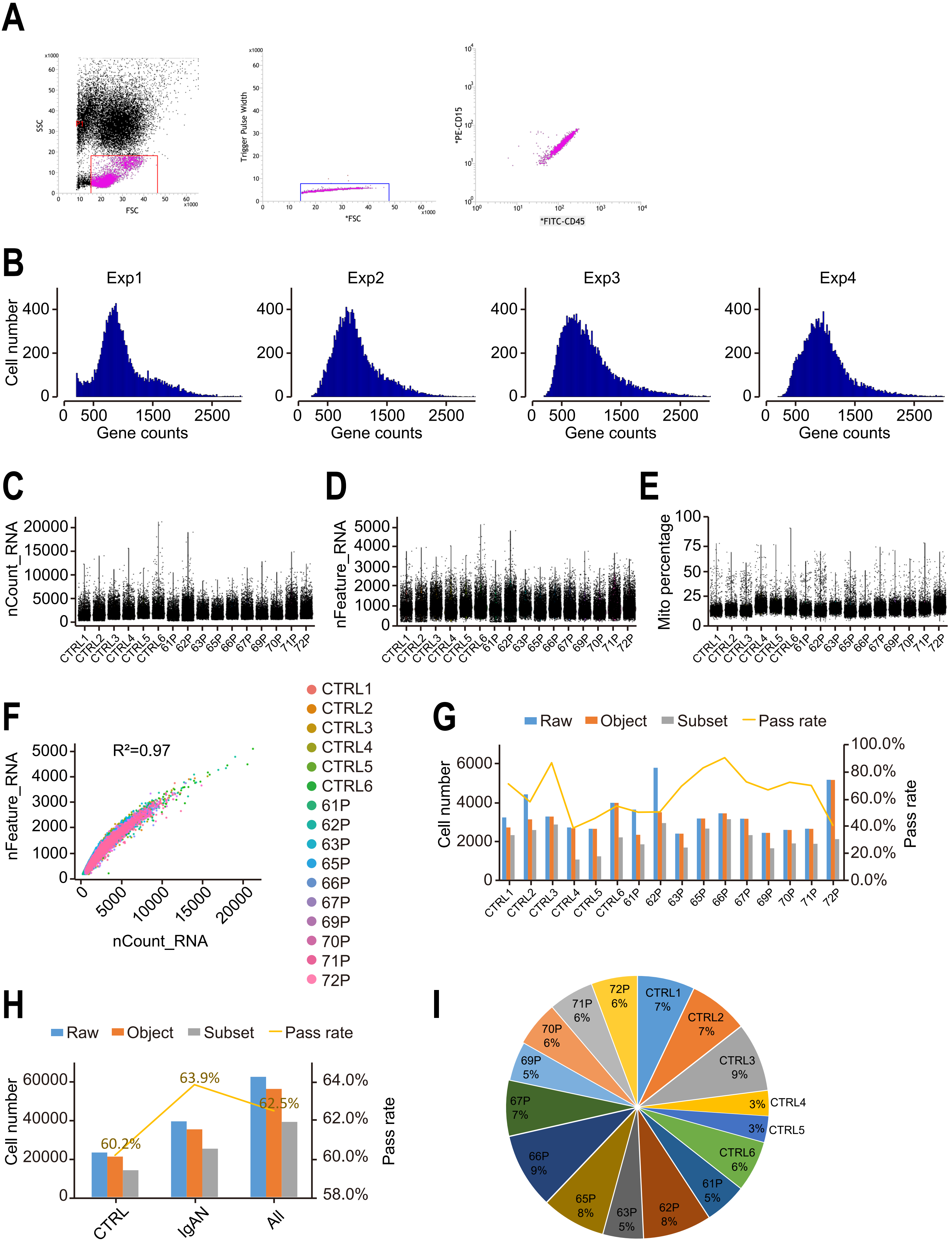

A
SSC
FSC
Trigger Pulse Width
*FSC
*PE-CD15
*FITC-CD45
B
Exp1
Exp2
Exp3
Exp4
Cell number
Gene counts
C
nCount_RNA
D
nFeature_RNA
E
Mito percentage
CTRL1
CTRL2
CTRL3
CTRL4
CTRL5
CTRL6
61P
62P
63P
65P
66P
67P
69P
70P
71P
72P
F
R²=0.97
nFeature_RNA
nCount_RNA
G
Raw
Object
Subset
Pass rate
Cell number
Pass rate
H
Raw
Object
Subset
Pass rate
60.2%
63.9%
62.5%
Cell number
Pass rate
CTRL
IgAN
All
I
CTRL1 7%
CTRL2 7%
CTRL3 9%
3% CTRL4
3% CTRL5
CTRL6 6%
61P 5%
62P 8%
63P 5%
65P 8%
66P 9%
67P 7%
69P 5%
70P 6%
71P 6%
72P 6%

Fig.S1

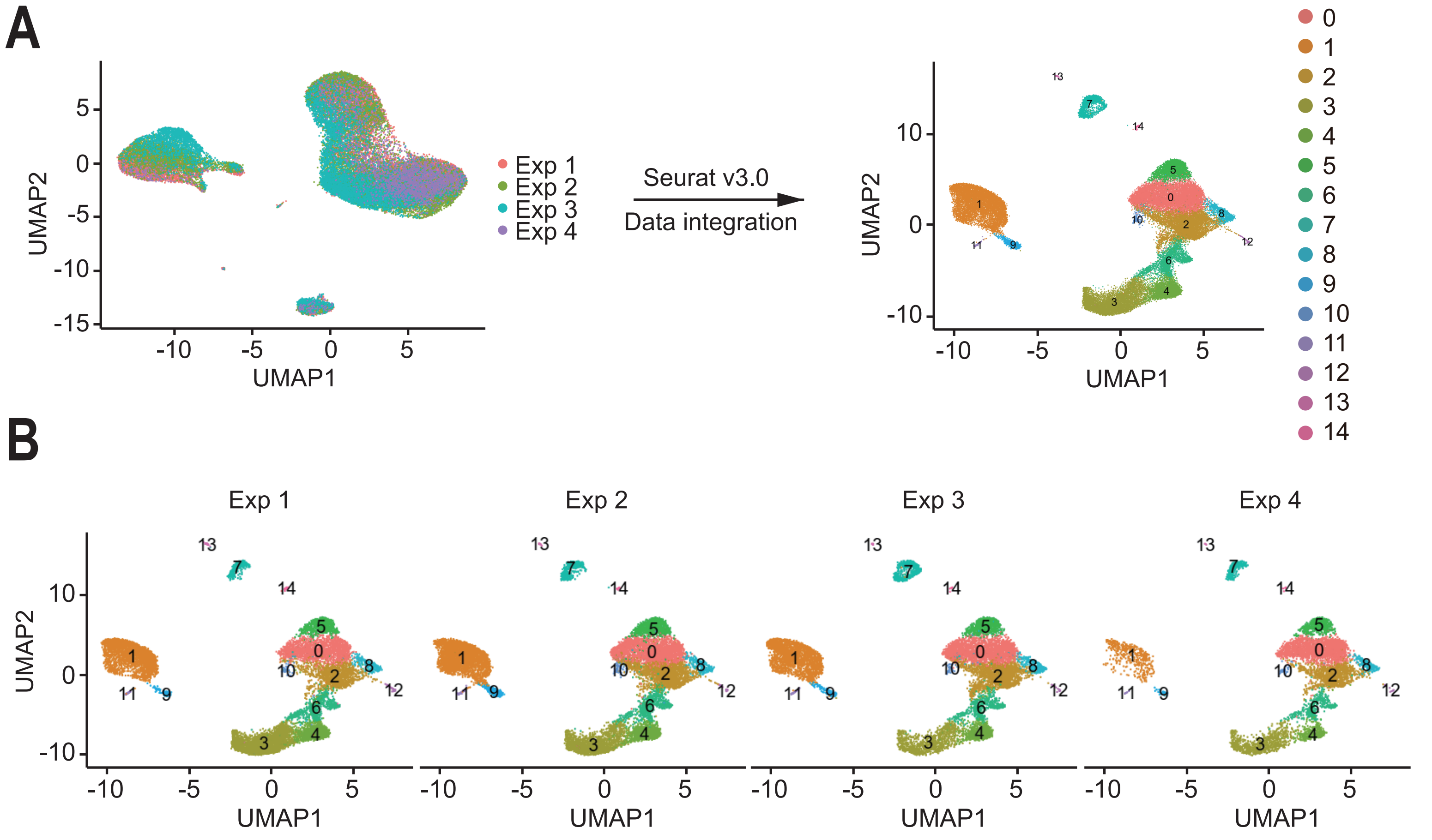
A
B
UMAP1
UMAP2
Exp 1
Exp 2
Exp 3
Exp 4
Seurat v3.0
Data integration
0
1
2
3
4
5
6
7
8
9
10
11
12
13
14

Fig.S2

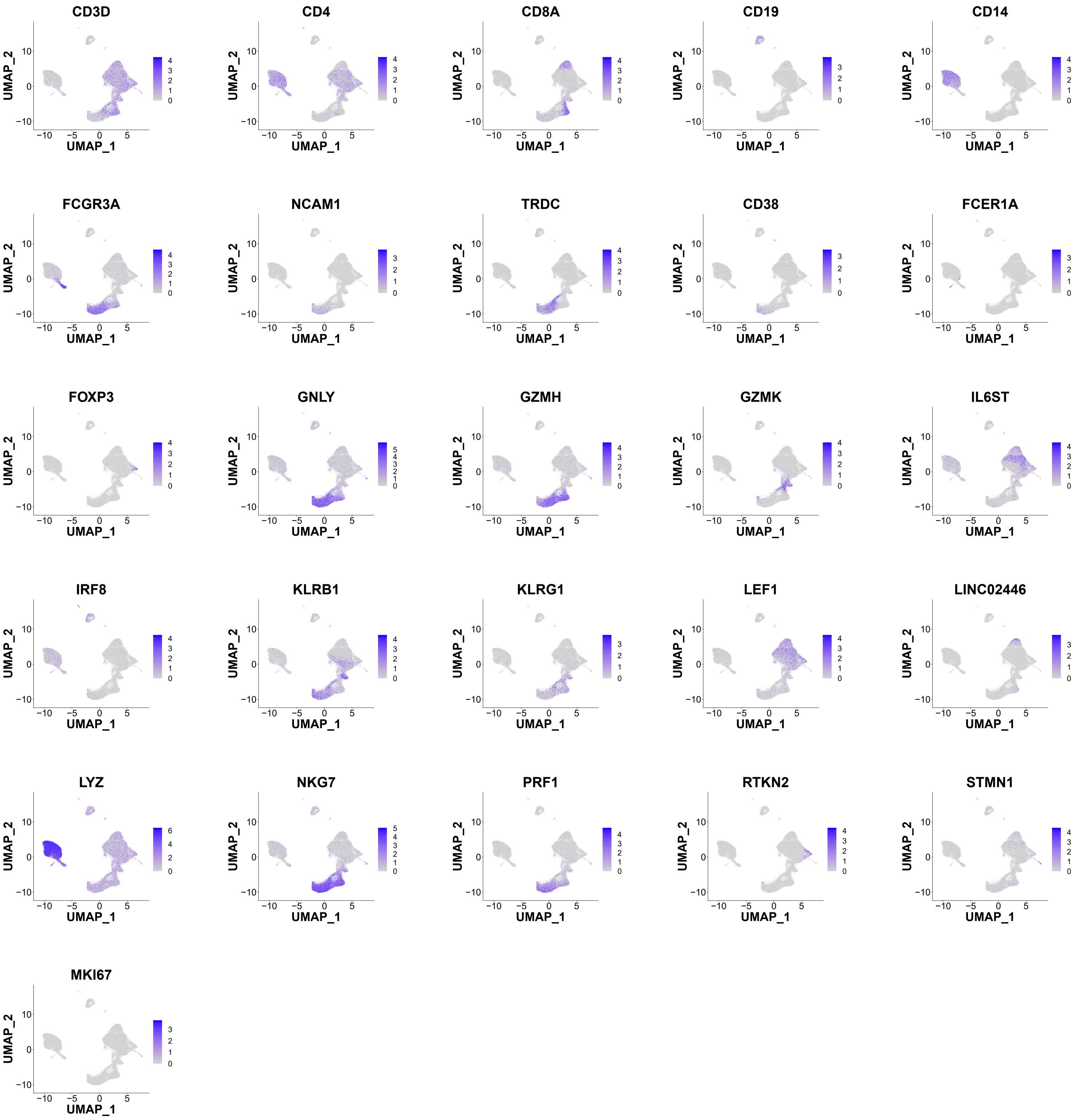

CD3D
CD4
CD8A
CD19
CD14
FCGR3A
NCAM1
TRDC
CD38
FCER1A
FOXP3
GNLY
GZMH
GZMK
IL6ST
IRF8
KLRB1
KLRG1
LEF1
LINC02446
LYZ
NKG7
PRF1
RTKN2
STMN1
MKI67
UMAP_1
UMAP_2

Fig.S3

A

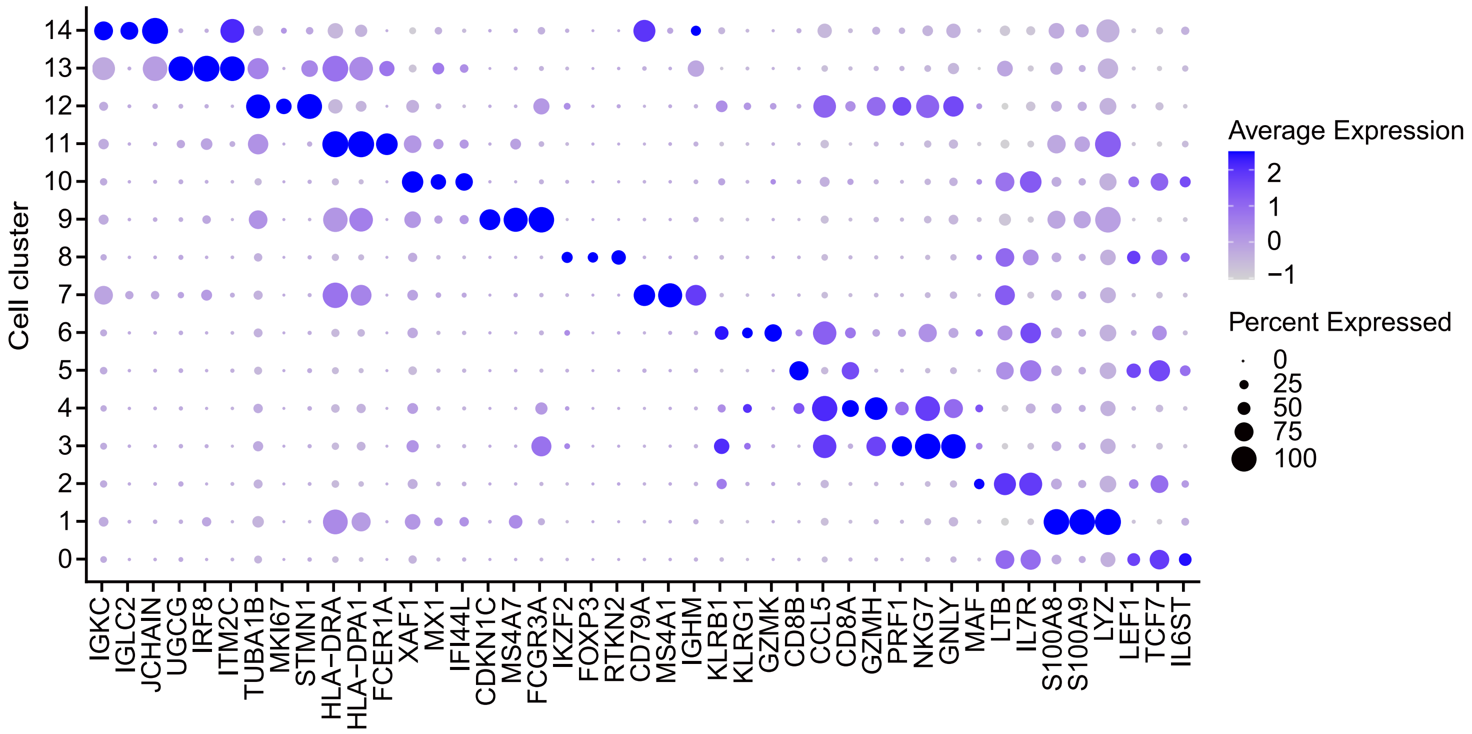
Cell cluster
14
13
12
11
10
9
8
7
6
5
4
3
2
1
0
IGKC
IGLC2
JCHAIN
UGCG
IRF8
ITM2C
TUBA1B
MKI67
STMN1
HLA−DRA
HLA−DPA1
FCER1A
XAF1
MX1
IFI44L
CDKN1C
MS4A7
FCGR3A
IKZF2
FOXP3
RTKN2
CD79A
MS4A1
IGHM
KLRB1
KLRG1
GZMK
CD8B
CCL5
CD8A
GZMH
PRF1
NKG7
GNLY
MAF
LTB
IL7R
S100A8
S100A9
LYZ
LEF1
TCF7
IL6ST
Average Expression
2
1
0
−1
Percent Expressed
0
25
50
75
100

Fig.S4

A

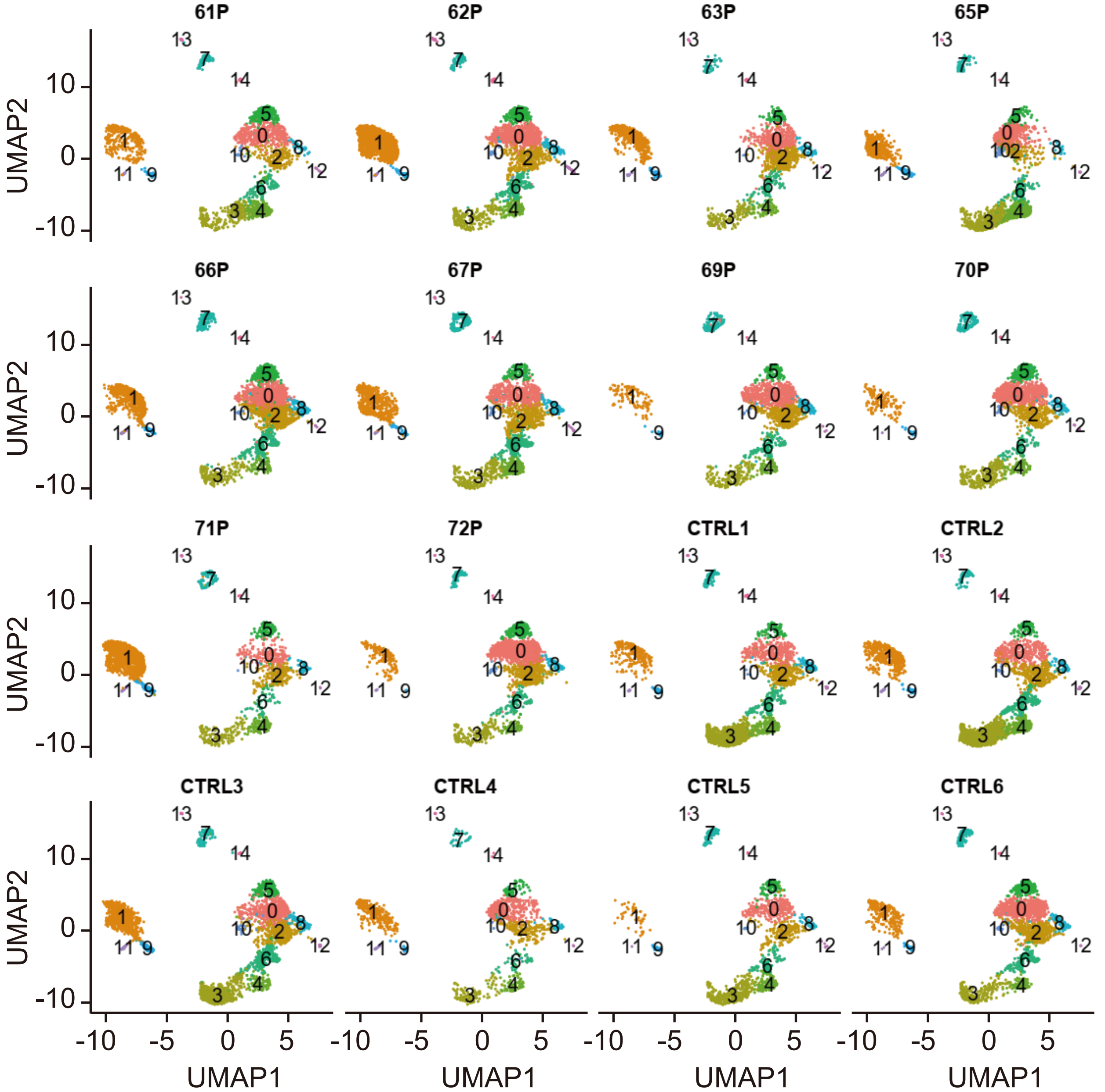
61P
62P
63P
65P
66P
67P
69P
70P
71P
72P
CTRL1
CTRL2
CTRL3
CTRL4
CTRL5
CTRL6
UMAP2
UMAP1

B

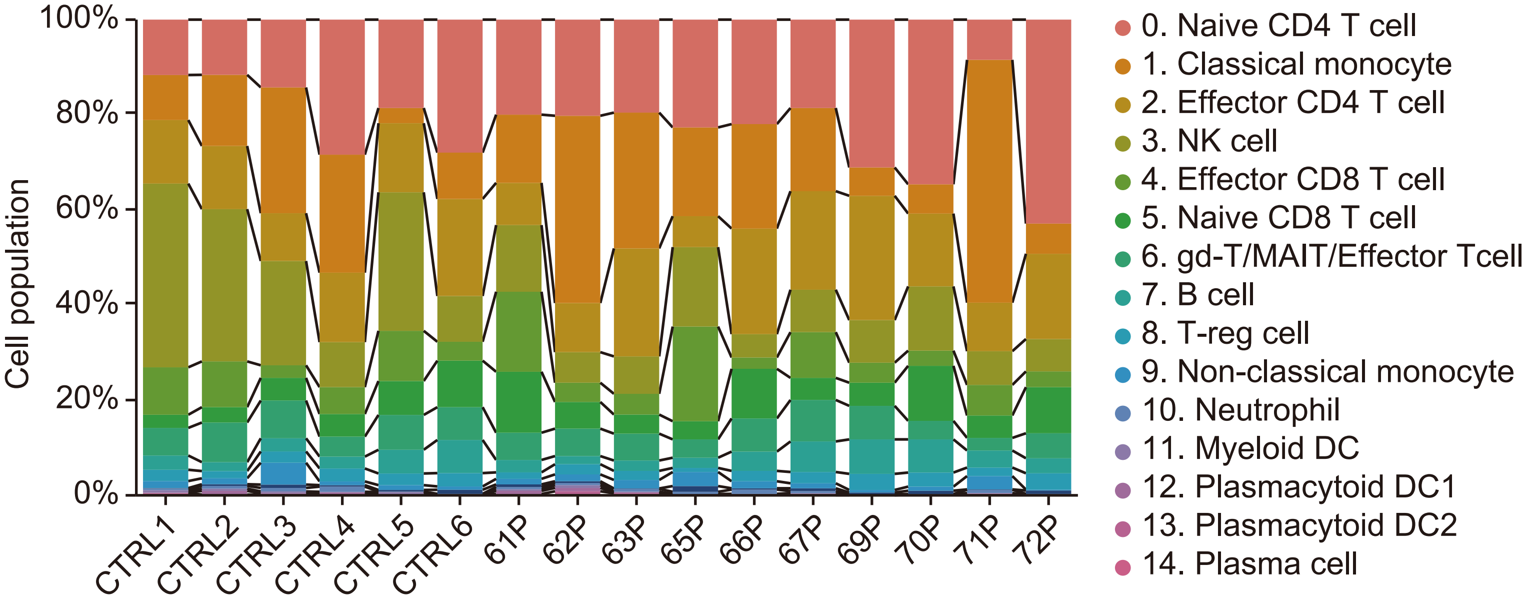
Cell population
100%
80%
60%
40%
20%
0%
CTRL1
CTRL2
CTRL3
CTRL4
CTRL5
CTRL6
61P
62P
63P
65P
66P
67P
69P
70P
71P
72P
0. Naive CD4 T cell
1. Classical monocyte
2. Effector CD4 T cell
3. NK cell
4. Effector CD8 T cell
5. Naive CD8 T cell
6. gd-T/MAIT/Effector Tcell
7. B cell
8. T-reg cell
9. Non-classical monocyte
10. Neutrophil
11. Myeloid DC
12. Plasmacytoid DC1
13. Plasmacytoid DC2
14. Plasma cell

Fig.S5

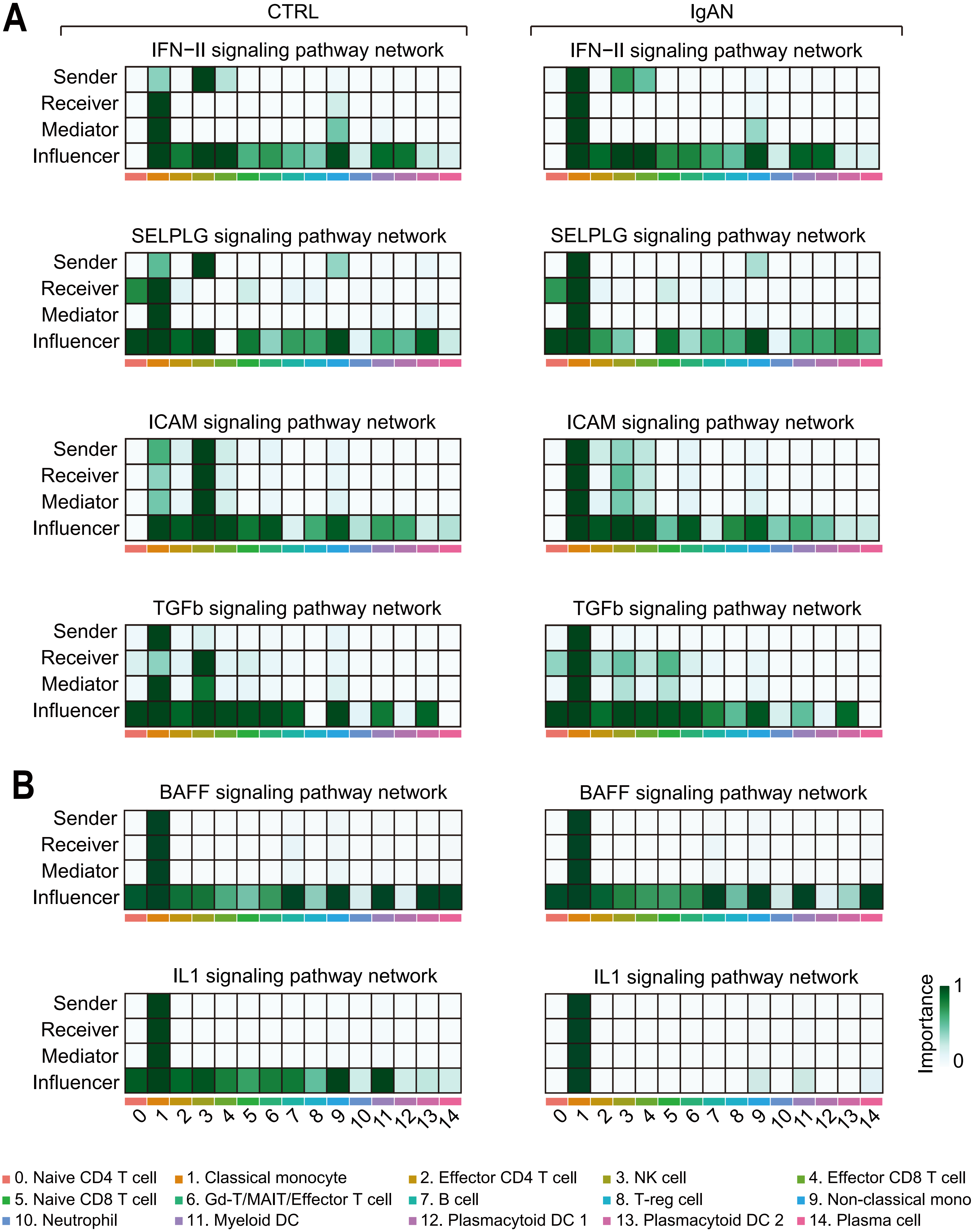

A
CTRL
IgAN
IFN−II signaling pathway network
SELPLG signaling pathway network
ICAM signaling pathway network
TGFb signaling pathway network
B
BAFF signaling pathway network
IL1 signaling pathway network
Sender
Receiver
Mediator
Influencer
0 1 2 3 4 5 6 7 8 9 10 11 12 13 14
Importance
1
0
0. Naive CD4 T cell
1. Classical monocyte
2. Effector CD4 T cell
3. NK cell
4. Effector CD8 T cell
5. Naive CD8 T cell
6. Gd-T/MAIT/Effector T cell
7. B cell
8. T-reg cell
9. Non-classical mono
10. Neutrophil
11. Myeloid DC
12. Plasmacytoid DC 1
13. Plasmacytoid DC 2
14. Plasma cell

Fig.S6

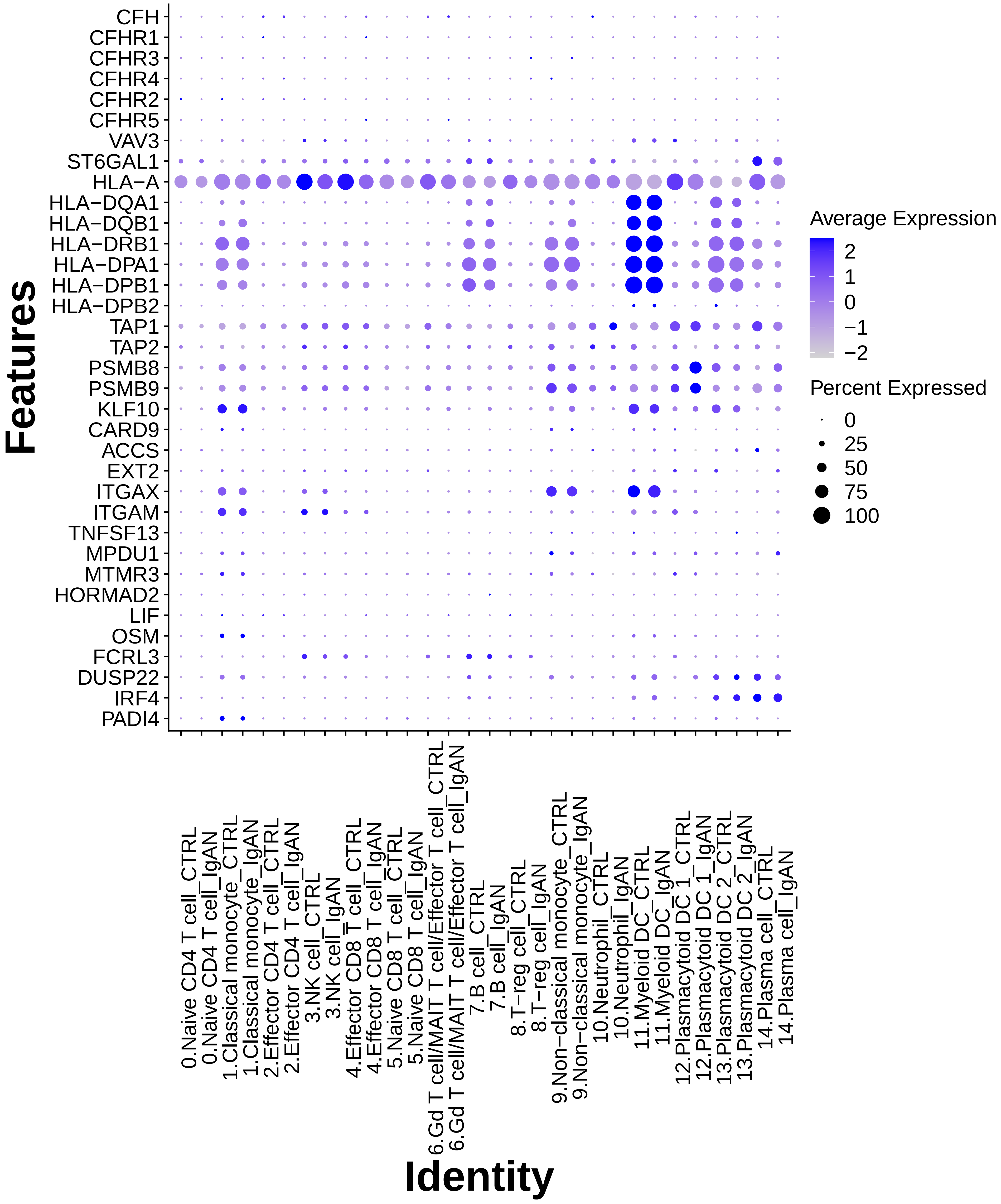
Features
CFH
CFHR1
CFHR3
CFHR4
CFHR2
CFHR5
VAV3
ST6GAL1
HLA−A
HLA−DQA1
HLA−DQB1
HLA−DRB1
HLA−DPA1
HLA−DPB1
HLA−DPB2
TAP1
TAP2
PSMB8
PSMB9
KLF10
CARD9
ACCS
EXT2
ITGAX
ITGAM
TNFSF13
MPDU1
MTMR3
HORMAD2
LIF
OSM
FCRL3
DUSP22
IRF4
PADI4
Average Expression
2
1
0
−1
−2
Percent Expressed
0
25
50
75
100
0.Naive CD4 T cell_CTRL
0.Naive CD4 T cell_IgAN
1.Classical monocyte_CTRL
1.Classical monocyte_IgAN
2.Effector CD4 T cell_CTRL
2.Effector CD4 T cell_IgAN
3.NK cell_CTRL
3.NK cell_IgAN
4.Effector CD8 T cell_CTRL
4.Effector CD8 T cell_IgAN
5.Naive CD8 T cell_CTRL
5.Naive CD8 T cell_IgAN
6.Gd T cell/MAIT T cell/Effector T cell_CTRL
6.Gd T cell/MAIT T cell/Effector T cell_IgAN
7.B cell_CTRL
7.B cell_IgAN
8.T−reg cell_CTRL
8.T−reg cell_IgAN
9.Non−classical monocyte_CTRL
9.Non−classical monocyte_IgAN
10.Neutrophil_CTRL
10.Neutrophil_IgAN
11.Myeloid DC_CTRL
11.Myeloid DC_IgAN
12.Plasmacytoid DC 1_CTRL
12.Plasmacytoid DC 1_IgAN
13.Plasmacytoid DC 2_CTRL
13.Plasmacytoid DC 2_IgAN
14.Plasma cell_CTRL
14.Plasma cell_IgAN
Identity
